# The eINTACT system dissects bacterial exploitation of plant osmosignalling to enhance virulence

**DOI:** 10.1038/s41477-022-01302-y

**Published:** 2022-12-22

**Authors:** Yuan You, Grzegorz Koczyk, Maria Nuc, Robert Morbitzer, Danalyn R. Holmes, Edda von Roepenack-Lahaye, Shiji Hou, Axel Giudicatti, Carine Gris, Pablo A. Manavella, Laurent D. Noël, Paweł Krajewski, Thomas Lahaye

**Affiliations:** 1 Department of General Genetics, Center for Plant Molecular Biology (ZMBP), Eberhard-Karls-University Tübingen, Tübingen, Germany; 2 Department of Biometry and Bioinformatics, Institute of Plant Genetics, Polish Academy of Sciences, Poznań, Poland; 3 Central Facilities - Analytics, ZMBP, Eberhard-Karls-University Tübingen, Tübingen, Germany; 4 State Key Laboratory of Agricultural Microbiology, Hubei Key Lab of Plant Pathology, Hubei Hongshan Laboratory, College of Plant Science and Technology, Huazhong Agricultural University, Wuhan, PR of China; 5 Instituto de Agrobiotecnología del Litoral (CONICET-UNL), Facultad de Bioquímica y Ciencias Biológicas, Universidad Nacional del Litoral, Santa Fe, Argentina; 6 Laboratoire des Interactions Plantes-Microbes-Environnement (LIPME), Université de Toulouse, INRAE, CNRS, Castanet-Tolosan, France

**Keywords:** Plant molecular biology, Biological models, Effectors in plant pathology

## Abstract

Bacteria inject effector proteins into host cells to manipulate cellular processes that promote disease. Since bacteria deliver minuscule amounts of effectors only into targeted host cells, it is technically challenging to capture effector-dependent cellular changes from bulk-infected host tissues. Here, we report a new technique called effector-inducible isolation of nuclei tagged in specific cell types (eINTACT), which facilitates affinity-based purification of nuclei from *Arabidopsis* plant cells that have received *Xanthomonas* bacterial effectors. Analysis of purified nuclei reveals that the *Xanthomonas* effector XopD manipulates the expression of *Arabidopsis* abscisic acid signalling-related genes and activates *OSCA1.1*, a gene encoding a calcium-permeable channel required for stomatal closure in response to osmotic stress. The loss of *OSCA1.1* causes leaf wilting and reduced bacterial growth in infected leaves, suggesting that *OSCA1.1* promotes host susceptibility. eINTACT allows us to uncover that XopD exploits host OSCA1.1/abscisic acid osmosignalling-mediated stomatal closure to create a humid habitat that favours bacterial growth and opens up a new avenue for accurately elucidating functions of effectors from numerous gram-negative plant bacteria in native infection contexts.

## Abstract (web summary)

eINTACT uncovers *Xanthomonas* bacterial exploitation of plant osmosignalling by its effector XopD to enhance virulence. This provides the basis for accurately elucidating functions of bacterial type-III effectors in natively infected plants.

## Main

Bacterial pathogens enter host plants via natural openings (for example, stomata or hydathodes) or wounds and subsequently multiply in the apoplastic space between cells or in the vessels^1,2^. To facilitate infection, virulent gram-negative bacteria inject effectors into host cells using a syringe-like multiprotein complex, the type III secretion system (T3SS)^3^. Inside host cells, effectors localize to specific subcellular compartments and manipulate host cellular processes to suppress host immunity and/or to create an environment that favours bacterial growth or dispersal^4,5^. Recent studies on bacterial pathogens suggest that establishing an aqueous apoplastic space in plants is critical for bacterial virulence^6^. Indeed, a few effectors were identified to promote disease by causing increased water levels in infected tissues, such as *Pseudomonas syringae* HopM1^6^, *Xanthomonas gardneri* AvrHah1^7^ and *Xanthomonas translucens* Tal8^8^. Due to the diversity of effectors in phylogenetically distinct bacterial genera, molecular mechanisms underlying effector-mediated plant susceptibility remain largely enigmatic.

**Extended Data Fig. 1:**
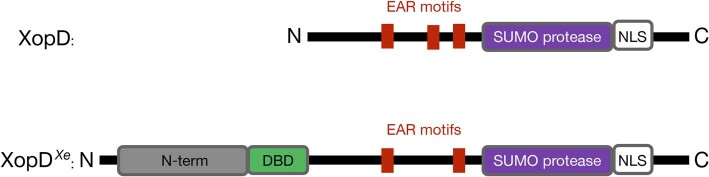
Structure analysis of XopD homologues. XopD of *Xanthomonas campestris* pv. *campestris* strain 8004 (XopD in our study) contains three plant-specific ethylene-responsive element binding factor-associated amphiphilic repression (EAR) motifs (red) at the N terminus, followed by a C-terminal cysteine protease domain (purple), and a plant nuclear localization sequence (NLS) at the C terminus. XopD^*Xe*^ from *X.* *euvesicatoria* strain 85-10 (*Xe*85-10) contains an N-terminal extension sequence (grey) and a putative DNA-binding helix-loop-helix domain (DBD, green) which are absent in XopD. XopD^*Xe*^ has only two EAR motifs.

*Xanthomonas campestris* pv. *campestris* strain 8004 (*Xcc*8004) is a leaf vascular pathogen that causes black rot disease in many *Brassica* crops and the model plant *Arabidopsis*^2^. One of its effector proteins, *Xanthomonas* outer protein D (XopD^*Xcc*8004^, in this study, XopD), is a nuclear-localized type III effector protein containing three N-terminal plant-specific ethylene-responsive element binding factor-associated amphiphilic repression (EAR) motifs that usually mediate transcriptional silencing in planta and a C-terminal cysteine protease domain^9^ (Extended Data Fig. 1). Intriguingly, two previous studies reported distinct activities of XopD in *Arabidopsis*^10,11^. One study showed that XopD promotes disease by suppressing early leaf necrosis, but does not affect bacterial growth in the infected *Arabidopsis* leaves^10^. Via its EAR motif-containing domain, XopD interacts with and stabilizes *Arabidopsis* DELLA proteins that are major transcriptional repressors of gibberellic acid(GA)-responsive genes^10^. Yet, the XopD–DELLA interaction does not cause detectable changes in the levels of GA-responsive transcripts^10^. Controversially, another study inferred an avirulence effect of XopD, as transgenic expression of XopD under control of a β-estradiol-inducible promoter in *Arabidopsis* triggered salicylic acid (SA)-dependent defence responses and suppressed bacterial growth^11^. Additionally, having small ubiquitin-like modifier (SUMO) protease activity, XopD has been found, in vitro, to interact with and deSUMOylate *Arabidopsis* HFR1, a transcription factor (TF) involved in phytochrome signalling^11^. Taken together, both previous studies lend themselves to suggest that XopD could modulate activity of plant TFs and host phytohormone signalling pathways. Nonetheless, the molecular mechanisms underlying the precise in planta functions of XopD remain inconclusive.

We sought to identify and characterize these ambiguous molecular mechanisms; however, current experimental approaches that aim to uncover in planta functions of bacterial effectors mostly ignore the cellular complexity of infected plant tissues. For example, after entering the apoplastic space in the mesophyll, bacterial pathogens inject their effectors only into plant cells that are accessible from the apoplastic lumen^3^. The neighbouring cells may receive some effectors that move from the cells directly receiving effectors through plasmodesmata; however, the concentration gradient is likely to be steep^12^. Conceivably, effector-induced changes in those neighbouring cells differ quantitatively depending on the functions and amounts of the specific effectors that move through plasmodesmata. Currently, most studies use bulk-infected host tissues as a starting material, consisting of a mixture of effector-recipient and non-recipient host cells, inevitably causing a dilution of effector-induced cellular changes, possibly to undetectable levels. Alternatively, transgenic expression of effector genes under constitutive or inducible promoters is commonly used to approximate their in planta functions. While simple and useful, the ectopic overexpression of effectors in plants disregards the fact that bacteria typically deliver only minuscule amounts of effector proteins into targeted host cells, possibly obscuring effector functions that are sensitive to dose-dependency, spatio-temporal infection dynamics and cellular specificity^4,13,14^. Therefore, there is an urgent need for new experimental techniques that allow the analysis of bacterial effector functions in the cells directly receiving effectors via T3SS-mediated delivery in natively infected host tissues.

## Results

### Purify nuclei from effector-recipient host cells by eINTACT

**Fig. 1:**
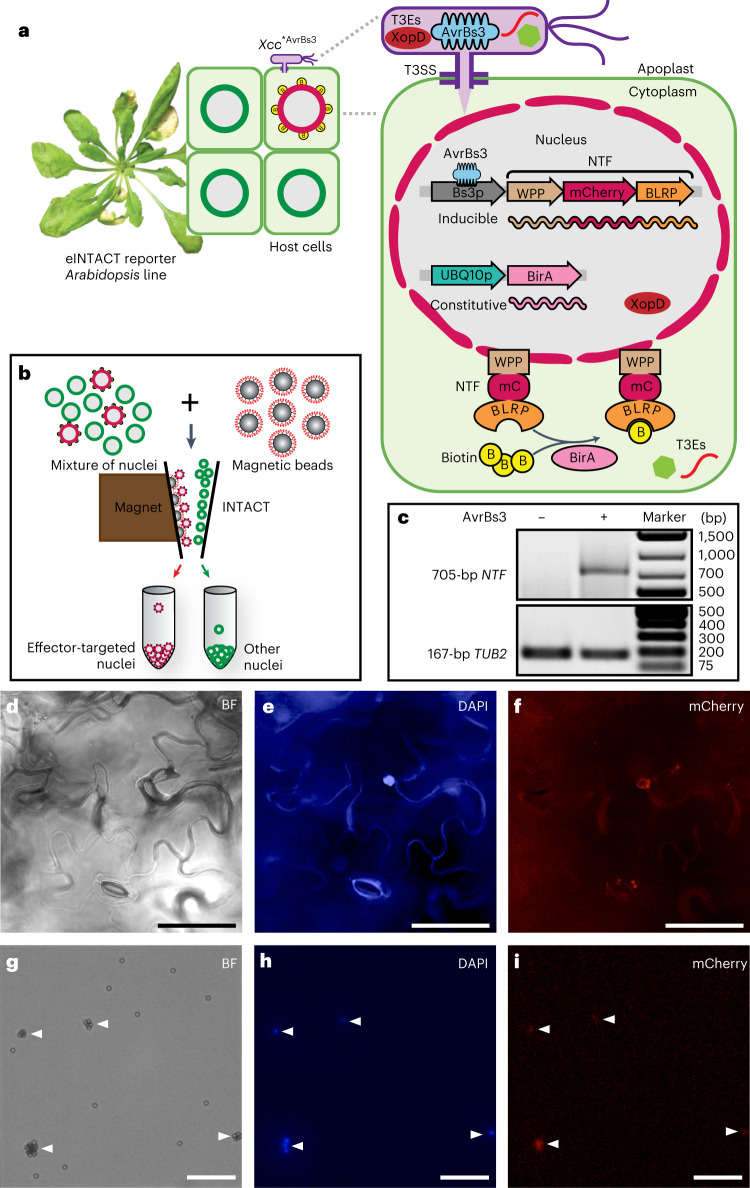
eINTACT enables the isolation of nuclei from effector-recipient host cells. **a**, Schematic of the *Arabidopsis-Xcc* eINTACT system (Supplementary Text). **b**, Isolation of the biotin-tagged nuclei using streptavidin-coated magnetic beads by affinity-based purification (INTACT method, Supplementary Text). **c**, Reverse transcription-polymerase chain reaction (RT-PCR) detection of *NTF* expression in Xcc^*vec1^ and Xcc^*AvrBs3^ infected leaves, at seven days postinfection (DPI). The expression of *TUB2* is used as a control. **d**–**f**, Microscopy images of leaf cells that have received Xcc^*AvrBs3^ effectors. Scale bar, 50 μm. **g**–**i** Microscopy images of eINTACT-purified nuclei (indicated with white triangles) from Xcc^*AvrBs3^ infected leaves. Scale bar, 100 μm. In microscopy images, BF indicates bright field (**d**,**g**); magnetic beads appear as white spheres (**g**); DNA stained with DAPI is shown in blue (**e**,**h**); and nuclear envelope domains tagged with red NTF are detected by mCherry fluorescence (**f**,**i**). Three repeats of each experiment (**c**–**i**) were performed independently with similar results.

To overcome the technical limitations of currently used methods for functional analysis of effectors in planta, we have developed a new technique, effector-inducible isolation of nuclei tagged in specific cell types (eINTACT), to selectively recover nuclei from effector-recipient cells of infected *Arabidopsis* plants (Fig. 1a,b). eINTACT relies on the transcriptional activation of a transgene-encoded biotinylated nuclear envelope-targeting protein (NTF)^15–17^ by effectors that bacteria inject into plant target cells. In the newly established eINTACT reporter *Arabidopsis* line, expression of the *NTF* transgene is under the control of the transcriptionally silent promoter of the pepper *Bs3* gene (*Bs3p*) that is activated by the bacterial transcription activator like (TAL) effector AvrBs3 from *Xanthomonas* *euvesicatoria* (*Xe*)^18^. The bacterial pathogen *Xcc* is used to deliver AvrBs3 into *Arabidopsis* cells to activate the NTF reporter. To generate an *Xcc*-derived strain that is fully virulent on *Arabidopsis*, named Xcc^*^, we deleted the bacterial genes encoding effectors AvrAC and XopAM that are known to trigger immunity in *Arabidopsis* accession Col-0^19^. We transformed Xcc^*^ with a pDSK vector containing *avrBs3* under the control of a constitutive promoter, yielding Xcc^*AvrBs3^. Therefore, Xcc^*AvrBs3^ will deliver AvrBs3 along with other Xcc^*^ effectors in the *Arabidopsis* eINTACT reporter line, leading to transcriptional activation of *Bs3p* and expression of the downstream *NTF* exclusively in effector-recipient host cells. A transgene-encoded, constitutively expressed biotin ligase mediates biotin labelling of the NTF protein, facilitating affinity purification of biotin-decorated nuclei from effector-targeted host cells using streptavidin-coated magnetic beads as described in the INTACT method^15–17^ (Fig. 1b).

**Extended Data Fig. 2:**
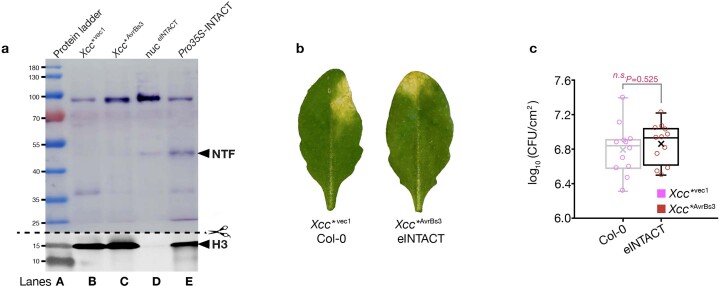
Validation of the eINTACT system. (**a**) Enrichment of the NTF protein in eINTACT-purified nuclei. NTF and H3 protein detection via Western blotting of the total nuclear protein extracts from Xcc^*vec1^-infected leaves (Xcc^*vec1^, lane **B**), the total nuclei (Xcc^*AvrBs3^, lane **C**) and eINTACT-purified nuclei (nuc^eINTACT^, lane **D**) from Xcc^*AvrBs3^*-*infected leaves. The blotted membrane was divided into two between 15 kDa and 25 kDa, according to size marks in the protein ladder (lane **A**). The upper part (25–180 kDa) was used for detecting biotinylated-NTF protein, using streptavidin alkaline phosphatase. The lower part (10–15 kDa) was used for detecting H3 protein using an anti-H3 antibody. The abundance of H3 protein serves as an internal control of the number of nuclei in each sample. A sample of total nuclei from mock-inoculated leaves of a *Pro35S*-INTACT reporter line, which overexpresses biotinylated-NTF under the control of the *35S* promoter, was used as a positive control (*Pro35S*-INTACT, lane **E**). The experiment was repeated twice independently with similar results. (**b**) A representative leaf of the eINTACT reporter line inoculated with Xcc^*AvrBs3^ and a representative wild-type Col-0 leaf inoculated with Xcc^*vec1^ at seven DPI. (**c**) A boxplot representing bacterial population density in n = 12 leaves of the eINTACT reporter line inoculated with Xcc^*AvrBs3^ and n = 12 wild-type Col-0 leaves inoculated with Xcc^*vec1^ at seven DPI. Horizontal lines from the top show maxima, upper quartile, median, lower quartile and minima values: cross marks show the mean values; and small circles show data points of individual biological replicates. The n = 12 leaves were collected from 4–6 different plants. Statistical significance is determined by two-sided unpaired *t*-test (eINTACT reporter line versus Col-0, *P* = 0.525). *n.s*., not significant (*P* > 0.05). Source data

As a proof-of-concept, we inoculated the eINTACT reporter line with Xcc^*AvrBs3^ and an isogenic strain containing the empty pDSK vector without *avrBs3* (Xcc^*vec1^). Due to *Xcc* being a vascular pathogen, in our study we inoculated the *Arabidopsis* leaves by wounding the central leaf vein with an *Xcc*-contaminated needle^19^. Seven days postinfection (DPI), we detected accumulation of *NTF* mRNA only in Xcc^*AvrBs3^, but not in Xcc^*vec1^, inoculated leaves (Fig. 1c), demonstrating that *NTF* expression is strictly AvrBs3 dependent. Furthermore, microscopic inspection of Xcc^*AvrBs3^ infected leaves revealed red-fluorescent NTF protein at the nuclear envelopes of effector-recipient cells (Fig. 1d–f), and western blotting showed enrichment of the NTF protein in eINTACT-purified nuclei (Extended Data Fig. 2a). Moreover, Xcc^*AvrBs3^ infected leaves of the eINTACT reporter line developed normal V-shaped chlorosis disease symptoms and hosted a similar amount of bacterial population as Xcc^*vec1^ infected wild-type Col-0 leaves (Extended Data Fig. 2b and c). Our results demonstrate that the eINTACT system is close to native infection conditions and provides an experimental basis to specifically recover nuclei of effector-recipient host cells from Xcc^*AvrBs3^ infected leaves (Fig. 1g–i).

### XopD changes host gene expression

**Fig. 2:**
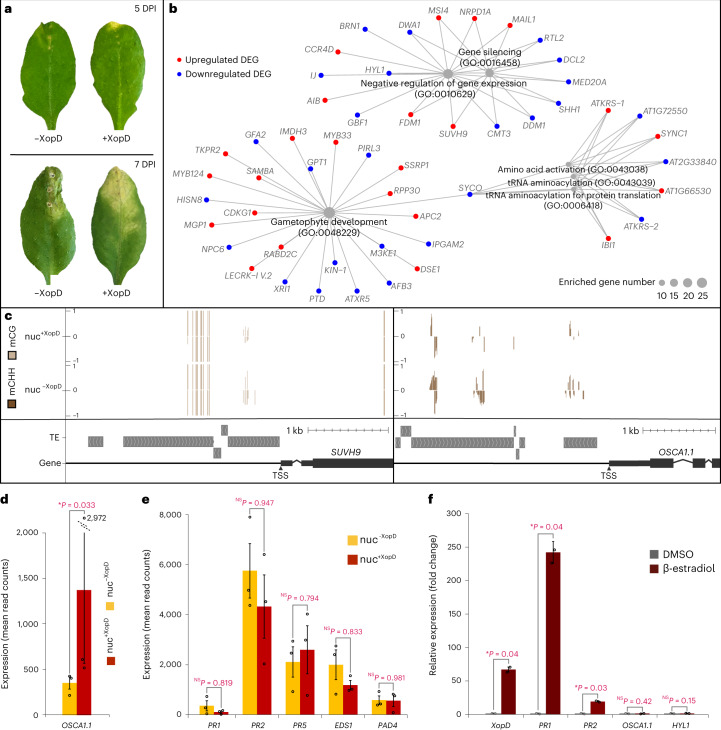
XopD-dependent host’s epigenetic transcriptional reprogramming. **a**, Representative *Arabidopsis* leaves infected with Xcc∆xopD^*AvrBs3^ (−XopD) and Xcc^*AvrBs3^ (+XopD) at five and seven DPI. **b**, Gene-concept network depicting connections between XopD-dependent DEGs and their significantly (FDR < 0.05; fold enrichment > 2) enriched biological GO terms. The DEGs that were not annotated by these GO terms were not shown. **c**, WashU Epigenome Browser snapshots showing mCG and mCHH methylation levels at *SUVH9* and *OSCA1.1* loci. Positive and negative bars indicate 5-methylcytosine levels of single cytosine on the Watson (+1) and Crick (−1) strands, respectively. The transposable elements (TEs) are shown as grey boxes. The transcription start site (TSS) is indicated with a brown triangle. **d**,**e**, The expression levels (mean read counts) of *OSCA1.1* (**d**) and *PR1*, *PR2*, *PR5*, *EDS1* and *PAD4* (**e**) in nuc^−XopD^ and nuc^+XopD^. Data are presented as mean values ± s.e.m. (error bars) from *n* = 3 independent biological replicates. DESeq2 *P* values are from Wald test corrected for multiple testing using the Benjamini–Hochberg method (nuc^−XopD^ versus nuc^+XopD^; *OSCA1.1*, *P* = 0.033; *PR1*, *P* = 0.819; *PR2*, *P* = 0.947; *PR5*, *P* = 0.794; *EDS1*, *P* = 0.833; *PAD4*, *P* = 0.981). *P < 0.05; NS, not significant (*P* > 0.05). **f**, Relative expression of *XopD*, *PR1*, *PR2*, *OSCA1.1* and *HYL1* after *XopD* was induced by β-estradiol in *Arabidopsis* seedlings, relative to their expression in DMSO-treated seedlings. The expression of *TUB2* is used as a control. Data are presented as mean values ± s.e.d. (error bars) from *n* = 2 independent biological replicates. Statistical significance is determined by a two-sided unpaired *t*-test (DMSO-treated seedlings versus β-estradiol-treated seedlings; *XopD*, *P* = 0.04; *PR1*, *P* = 0.04; *PR2*, *P* = 0.03; *OSCA1.1*, *P* = 0.42; *HYL1*, *P* = 0.15). *P < 0.05; NS, not significant (*P* > 0.05). Small circles indicate data points of individual biological replicates (**d**–**f**). Source data

To study in planta functions of the nuclear-targeted effector XopD, we inoculated leaves of the eINTACT reporter line with Xcc^*AvrBs3^ expressing XopD or the corresponding *xopD*-deletion mutant Xcc∆xopD^*AvrBs3^. At five DPI, Xcc∆xopD^*AvrBs3^ infected leaves started to show wilting lesions, which progressed into dehydrated and necrotic symptoms at the late stage of infection (seven DPI); while Xcc^*AvrBs3^ infected leaves developed yellow, V-shaped disease symptoms, indicating that XopD alters disease symptoms and promotes bacterial proliferation in the infected leaves (Fig. 2a). Therefore, with our eINTACT system, we purified nuclei that either received XopD (nuc^+XopD^) or did not receive XopD (nuc^−^^XopD^) from Xcc^*AvrBs3^ and Xcc∆xopD^*AvrBs3^ infected leaves, respectively, at five DPI. Differential analysis of nuc^+XopD^ versus nuc^−^^XopD^ thus provided the basis to uncover XopD-triggered changes in nuclei of effector-recipient host cells.

Comparison of nuclear transcriptomes in nuc^+XopD^ versus nuc^−^^XopD^ by RNA sequencing identified 924 differentially expressed genes (DEGs) (Supplementary Table 1). Gene ontology (GO) analysis revealed that these DEGs were significantly enriched for transcripts involved in ‘gene silencing’, ‘negative regulation of gene expression’, ‘gametophyte development’ and ‘tRNA aminoacylation’ functions (Fig. 2b and Supplementary Table 2). Specifically, GO analysis revealed 13 DEGs encoding important components of either RNA-directed DNA methylation or small RNA silencing pathways, which are predominantly known to affect gene silencing via epigenetic regulation and RNA interference, respectively (Table 1). This suggests that XopD-dependent changes in host gene expression could, at least in part, be regulated at the epigenetic level.

**Table 1:** XopD-affected genes involved in ‘gene silencing’

ID	Gene	Full name	Expression change^a,b^	Annotation
*AT1G63020*	*NRPD1A*	*NUCLEAR RNA POLYMERASE D 1A*	5.17	The unique, largest subunit of Pol IV required for siRNA synthesis^20^.
*AT2G19520*	*MSI4*	*MULTICOPY SUPPRESSOR OF IRA1 4*	3.89	Interacts with SUVH9 and is involved in RNA-directed DNA methylation by facilitating the recruitment of Pol V to chromatin^56^.
*AT4G13460*	*SUVH9*	*SU(VAR)3–9 HOMOLOGUE 9*	3.77	A SET-domain containing histone methyltransferase required for Pol V-mediated de novo methylation^20^.
*AT1G15910*	*FDM1*	*FACTOR OF DNA METHYLATION 1*	2.96	A SGS3-like protein required for de novo DNA methylation and siRNA accumulation^20^.
*AT2G25010*	*MAIL1*	*MAIN-LIKE 1*	2.15	A nuclear-localized aminotransferase-like protein that regulates silencing pathway independent of DNA methylation and short interfering RNAs^57^.
*AT2G28230*	*MED20A*	*MEDIATOR 20A*	−6.28	Involved in miRNA biogenesis by recruiting Pol II to promoters of miRNA genes^58^.
*AT5G66750*	*DDM1*	*DECREASED DNA METHYLATION 1*	−5.71	A chromatin remodeller required for the maintenance of DNA methylation^20^.
*AT3G20420*	*RTL2*	*RNASE THREE-LIKE PROTEIN 2*	−4.32	A double-stranded RNA binding ribonuclease III, involved in siRNA production^59^.
*AT1G69770*	*CMT3*	*CHROMOMETHYLASE 3*	−4.16	A chromomethylase involved in non-CG methylation^20^.
*AT3G03300*	*DCL2*	*DICER-LIKE 2*	−3.22	A Dicer-like protein involved in siRNA production^20^.
*AT1G15215*	*SHH1*	*SAWADEE HOMEODOMAIN HOMOLOGUE 1*	−3.16	A homeodomain protein that binds to methylated H3K9 and recruits Pol IV^20^.
*AT2G19430*	*DWA1*	*DDB1-BINDING WD40 PROTEIN HYPERSENSITIVE TO ABA 1*	−4.16	A protein with a DWD motif involved in the production of secondary siRNA^60^.
*AT1G09700*	*HYL1*	*HYPONASTIC LEAVES 1*	−2.41	A core miRNA biogenesis factor involved in mRNA cleavage^29,31^.

^a^Log_2_(fold change) in the comparison of nuc^+XopD^ versus nuc^−^^XopD^

^b^Positive value, the expression is increased in nuc^+XopD^; negative value, the expression is decreased in nuc^+XopD^

**Extended Data Fig. 3:**
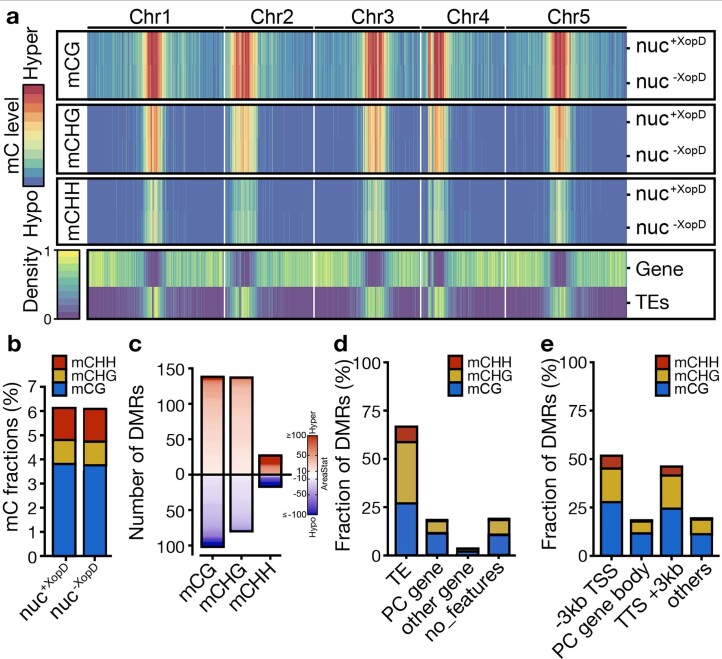
DNA-methylation patterns in nuc^+XopD^ and nuc^−XopD^. (**a**) Heatmaps showing mC levels in each context and density of genes and transposable elements (TEs), within 100 kb bins over the entire genome. Maximum mC levels are 0.88 (mCG), 0.50 (mCHG), 0.17 (mCHH). (**b**) Global levels of DNA methylation in nuc^+XopD^ and nuc^−XopD^. (**c**) Numbers of each type of differentially methylated regions (DMRs) in comparison of nuc^+XopD^ versus nuc^−XopD^. DMRs are sorted by AreaStat values (10–100, DSS test statistic) in heatmaps. (**d**) Fractions of DMRs overlapping with TEs, protein-coding (PC) genes, other genes and no genomic features. Some DMRs belong to multiple categories. (**e**) Overlapping between 3-kb proximal regions of PC genes and DMRs. Others refer to DMRs that do not overlap with 3-kb proximal gene regions. TSS, transcription start site; TTS, transcription termination site. Some DMRs belong to multiple categories.

We therefore investigated XopD-dependent changes in genome-wide DNA methylation on cytosines (mC) (Extended Data Fig. 3 and Supplementary Table 3) and their correlation with transcriptional changes in nuc^+XopD^ versus nuc^−^^XopD^. We identified 19 DEGs that correlated with differentially methylated regions (DMRs) within their 3-kb proximal promoter regions (Supplementary Table 4). These 19 DEGs include, for example, *SUVH9* encoding a histone methyltransferase^20^ and*OSCA1.1* encoding a Ca^2+^-permeable channel protein involved in osmotic stress-induced stomatal closure in *Arabidopsis*^21^. XopD caused reduced mCG levels in the *SUVH9* promoter and reduced mCHH levels in the *OSCA1.1* promoter (Fig. 2c). Since increased promoter methylation usually correlates with reduced transcription of the downstream genes^20^, our results indicate that elevated *SUVH9* and *OSCA1.1* transcript levels (Table 1 and Fig. 2d) are likely the consequence of XopD-induced demethylation of their promoters (Fig. 2c).

### eINTACT is superior for revealing XopD functions in planta

A previous study showed that in planta expression of XopD under control of a β-estradiol-inducible promoter triggered an SA-mediated defence response in *Arabidopsis*^11^. By contrast, our investigation, which is based on a native infection scenario where the bacterial pathogen *Xcc* injects XopD into targeted host cells, did not uncover changes in transcript abundance of key SA-dependent genes (Fig. 2e and Supplementary Table 5). The discrepancies between the two studies are not limited to SA-related genes. In addition, we found no overlap between XopD-dependent DEGs in our eINTACT data and genes whose transcript levels were changed after XopD was induced by β-estradiol^11^. Case in point, both *OSCA1.1* and *HYL1*, which had XopD-dependent expression changes in effector-recipient cells (Fig. 2d and Table 1), did not change in expression after the β-estradiol-induction of XopD in *Arabidopsis* seedlings (Fig. 2f).

During infection, Xcc^*AvrBs3^ T3SS-mediated delivery results in trace amounts of XopD only in host cells that are targeted by bacteria^4,13,14^. However, β-estradiol-induced ectopic expression causes high XopD levels in all plant cells, which conceivably exacerbates its in planta activity, resulting in the misregulation of gene expression and plant physiology in a way that does not mirror a natural infection. As such, it was previously shown that induced expression of XopD in *Arabidopsis* caused localized necrotic spots and cell death in leaves^11^, while native bacteria inject XopD to suppress leaf necrosis (Fig. 2a). These observed discrepancies advocate the need of studying effector functions in an infection system that is as close to native conditions as possible.

**Extended Data Fig. 4:**
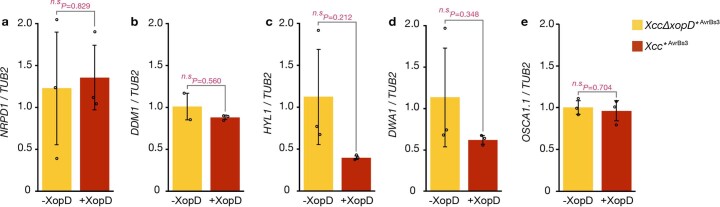
RT-qPCR analysis of relative expression of *NRPD1A*, *DDM1*, *HYL1*, *DWA1* and *OSCA1.1* in Xcc∆xopD^*AvrBs3^ (−XopD) and Xcc^*AvrBs3^ (+XopD) inoculated leaves of the eINTACT reporter line at five DPI. (**a**–**e**) Data are presented as mean values +/− s.d. (error bars) from n = 2 or 3 independent biological replicates. Small circles show data points of individual biological replicates. Statistical significance is determined by two-sided unpaired *t*-test (Xcc∆xopD^*AvrBs3^ inoculated leaves versus Xcc^*AvrBs3^ inoculated leaves; *NRPD1A*, *P* = 0.829; *DDM1*, *P* = 0.560; *HYL1*, *P* = 0.212; *DWA1*, *P* = 0.348; *OSCA1.1*, *P* = 0.704). *n.s*., not significant (*P* > 0.05). Source data

To clarify if our effector-recipient cell-specific study indeed improved the discovery of XopD-dependent expression changes, we performed differential gene expression analysis using whole-leaf tissues infected with Xcc^*AvrBs3^ versus Xcc∆xopD^*AvrBs3^ as starting materials (Extended Data Fig. 4). We did not observe changes of *NRPD1A*, *DDM1*, *HYL1*, *DWA1* and *OSCA1.1* transcript levels in whole infected leaf tissues (Extended Data Fig. 4); even though some of these changes followed similar trends as the comparisons of nuc^+XopD^ versus nuc^−^^XopD^ (Table 1), the differences were much smaller and not significant (Extended Data Fig. 4). These observations indicate that XopD-dependent expression changes uncovered in effector-recipient nuclei are diluted to non-significant levels if analysed in bulk-infected leaf tissues, demonstrating the power of our eINTACT approach.

### *OSCA1.1* is required for susceptibility and for XopD function

Since *OSCA1.1* was found to have a XopD-dependent increase in expression (Fig. 2d), it raises the question of the biological relevance of *OSCA1.1* induction to *Xcc* virulence.

**Fig. 3:**
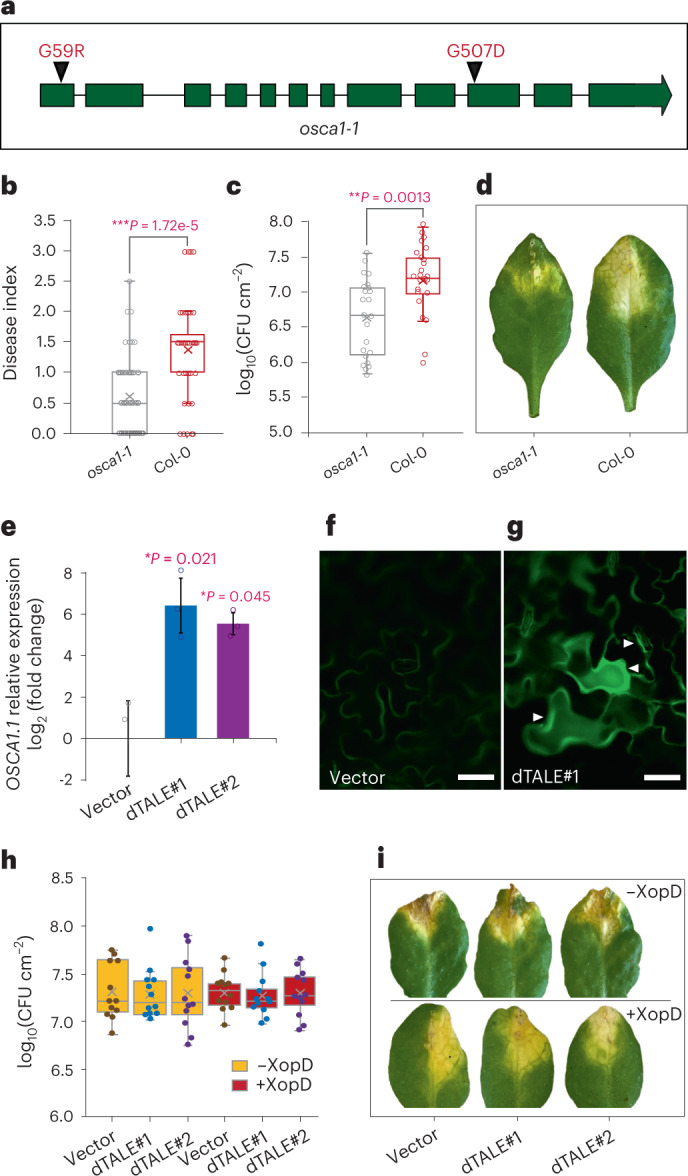
OSCA1.1 is required for bacterial virulence. **a**, Gene model of OSCA1.1 showing the positions of the two amino acid substitutions in the *osca1-1* mutant. **b**, A boxplot representing disease index scores (0, no symptoms; 0.5 to 1.5, weak chlorosis; 2 to 3, strong chlorosis) from *n* = 40 each of *osca1-1* or Col-0 leaves infected with Xcc^*^ from eight individual plants, at seven DPI. Statistical significance is determined by a two-sided unpaired *t*-test (*osca1-1* versus Col-0, *P* = 1.72e-5). ***P < 0.001. **c**, A boxplot representing bacterial population density in *n* = 23 *osca1-1* or *n* = 24 Col-0 leaves infected with Xcc^*^ from eight different plants, at seven DPI. CFU, colony-forming units. Statistical significance is determined by a two-sided unpaired *t*-test (*osca1-1* versus Col-0, *P* = 0.0013). **P < 0.01. **d**, Representative Xcc^*^ inoculated *osca1-1* and Col-0 leaves at nine DPI. **e**, Relative expression of *OSCA1.1* in Col-0 leaves infiltrated with Xcc∆xopD^*dTALE#1^ (dTALE#1) and Xcc∆xopD^*dTALE#2^ (dTALE#2), relative to its expression in Xcc∆xopD^*vec2^ (vector) infiltrated leaves, at one DPI. The expression of *TUB2* is used as a control. Data are presented as mean values ± s.d. (error bars) from *n* = 3 independent biological replicates. Small circles, data points of individual biological replicates. Statistical significance is determined by a two-sided unpaired *t*-test (dTALE#1 versus vector, *P* = 0.021; dTALE#2 versus vector, *P* = 0.045). *P < 0.05. **f**,**g**, Microscopy images of the epidermal pavement cells and guard cells expressing *OSCA1.1-GFP* at the plasma membrane in Xcc∆xopD^*vec2^ (vector) (**f**) and Xcc∆xopD^*dTALE*#*1^ (dTALE#1) (**g**) inoculated leaves of the *ProOSCA1.1:OSCA1.1-GFP* transgenic line at five DPI. The effector-recipient cells are indicated with white triangles. The experiments were repeated twice independently with similar results. **h**, A boxplot representing bacterial population density in Col-0 leaves inoculated with Xcc∆xopD^*^ (−XopD) and Xcc^*^ (+XopD) strains that deliver empty vector (vector), dTALE#1 or dTALE#2, at seven DPI. For each bacterial strain, *n* = 12 infected leaves from four individual plants were examined. Differences between bacterial populations of all bacterial strains were not significant (one-way ANOVA test). **i**, Representative leaves used in (**h**). On the boxplots (**b**,**c**,**h**), horizontal lines from the top show maxima, upper quartile, median, lower quartile and minima values; cross marks show the mean values; and small circles show data points of individual biological replicates. Source data

**Extended Data Fig. 5:**
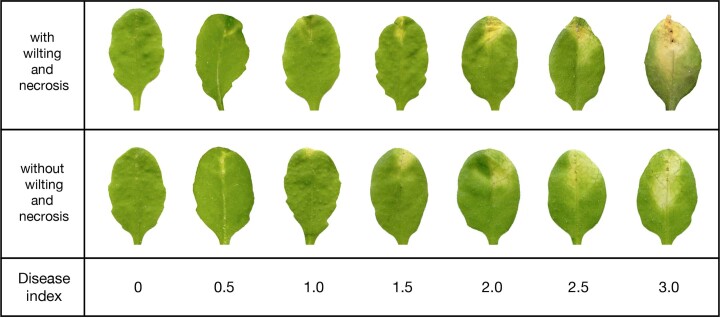
The disease index scores describing the degrees and sizes of chlorotic symptoms. This index system is modified from Guy et al.^19^: 0, no symptoms; 0.5 to 1.5, weak chlorosis; 2 to 3, strong chlorosis. The degree of wilting and necrosis was not considered in this index system.

**Extended Data Fig. 6:**
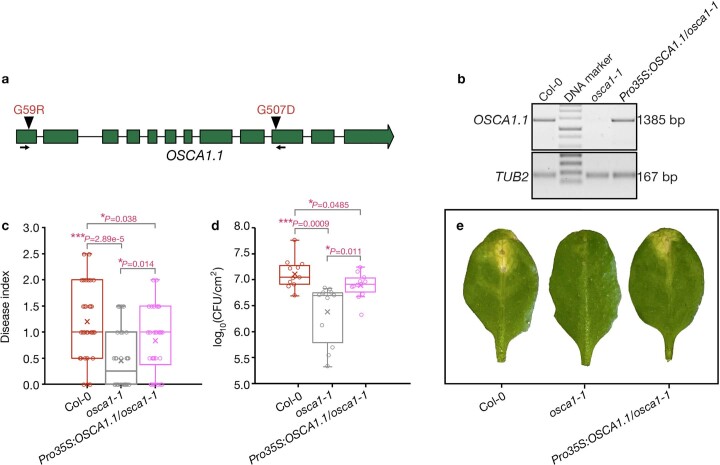
Constitutive expression of *OSCA1.1* partially complements the *osca1-1* disease resistance phenotype. (**a**) Gene model of *OSCA1.1* showing the positions of wild-type *OSCA1.1*-specific primer pairs that contain the nuclear acids mutated in *osca1-1* at their 3’ ends. (**b**) Semi-quantitative RT–PCR detecting wild-type *OSCA1.1* transcripts in Col-0, *osca1-1* and *Pro35S:OSCA1.1/osca1-1* plants. The experiment was repeated twice independently with similar results. (**c**) A boxplot representing disease index scores (0, no symptoms; 0.5 to 1.5, weak chlorosis; 2 to 3, strong chlorosis) from n = 30 each of Col-0, *osca1-1* or *Pro35S:OSCA1.1/osca1-1* leaves infected with Xcc^*^ from 8 individual plants at seven DPI. Statistical significance is determined by two-sided unpaired *t*-test (Col-0 versus *osca1-1*, *P* = 2.89e-5; Col-0 versus *Pro35S:OSCA1.1/osca1-1*, *P* = 0.038; *osca1-1* versus *Pro35S:OSCA1.1/osca1-1*, *P* = 0.014). *P < 0.05, ***P < 0.001. (**d**) A boxplot representing bacterial population density in n = 12 each of Col-0, *osca1-1* or *Pro35S:OSCA1.1/osca1-1* leaves infected with Xcc^*^ from 4 different plants at seven DPI. CFU, colony-forming units. Statistical significance is determined by two-sided unpaired *t*-test (Col-0 versus *osca1-1*, *P* = 0.0009; Col-0 versus *Pro35S:OSCA1.1/osca1-1*, *P* = 0.011; *osca1-1* versus *Pro35S:OSCA1.1/osca1-1*, *P* = 0.0485). (**c**, **d**) On these boxplots, horizontal lines from the top show maxima, upper quartile, median, lower quartile and minima values; cross marks show the mean values; and small circles show data points of individual biological replicates. (**d**) Representative Xcc^*^ inoculated Col-0, *osca1-1* or *Pro35S:OSCA1.1/osca1-1* leaves at seven DPI. Source data

OSCA1.1 is a plasma membrane-localized Ca^2+^-permeable channel protein that acts as an osmosensor in *Arabidopsis*^21^. Mutations in *OSCA1.1* cause impaired Ca^2+^ increases and failure of stomatal closure upon osmotic stress^21^. To assess whether or not OSCA1.1 plays a role in *Xcc* infection, we obtained *osca1-1*, a null mutant containing two amino acid substitutions resulting in loss of OSCA1.1 function^21^ (Fig. 3a). We inoculated the *osca1-1* plants and wild-type (Col-0) control plants with Xcc^*^ expressing XopD. We observed that the infected *osca1-1* leaves had a significantly lower degree of V-shaped chlorosis disease symptoms and hosted reduced bacterial population when compared to the infected Col-0 controls (Fig. 3b,c and Extended Data Fig. 5). At the late stage of infection (nine DPI), infected *osca1-1* leaves showed tissue wilting and necrosis, in contrast to chlorotic disease symptoms in the infected Col-0 leaves (Fig. 3d). Additionally, we inoculated Xcc^*^ bacteria into the leaves of a previously established transgenic line expressing *OSCA1.1* under the control of a *35S* promoter in the *osca1-1* mutant background (*Pro35S:OSCA1.1/osca1-1*), where constitutive expression of *OSCA1.1* complements the impaired osmotic Ca^2+^ signalling in the *osca1-1* mutant in response to osmotic stress^21^. We observed that the degree of disease symptoms and the amount of bacterial population in infected *Pro35S:OSCA1.1/osca1-1* leaves were significantly higher when compared to the infected *osca1-1* leaves but still lower when compared to the infected Col-0 controls (Extended Data Fig. 6). In summary, these results demonstrate that *OSCA1.1* is important for bacterial disease and XopD-dependent suppression of leaf necrosis, and transcriptional activation of *OSCA1.1* in bacterial effector-targeted cells may be required for optimal host susceptibility.

**Extended Data Fig. 7:**
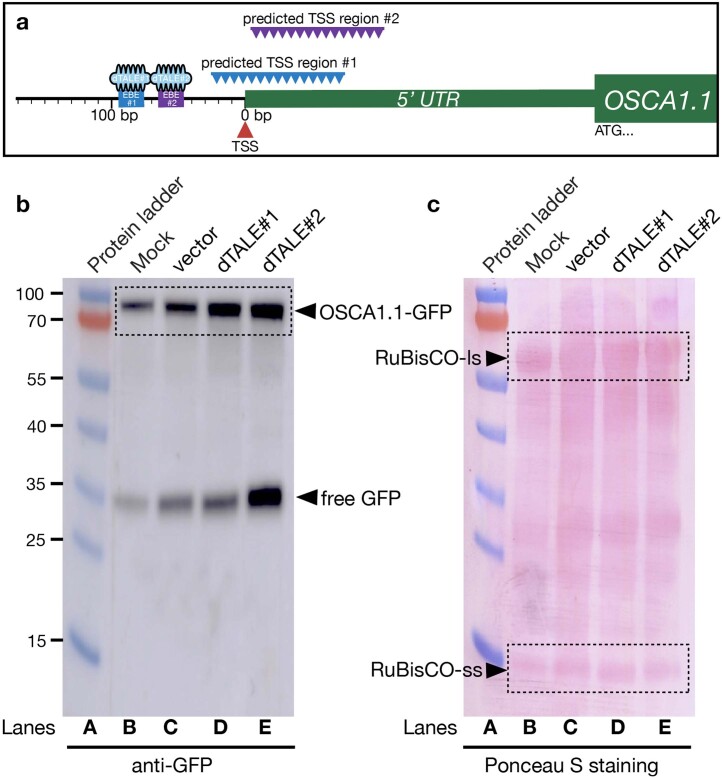
dTALEs induce *OSCA1.1* overexpression. (**a**) Graphical description showing the positions of EBEs of dTALE#1 and dTALE#2 in the *OSCA1.1* gene promoter and the predicted regions (80–150 bp downstream the EBEs) of the TSSs of dTALE-activated transcripts. (**b**) Immunoblotting detection of OSCA1.1-GFP protein in Mock (lane **B**), Xcc∆xopD^*vec2^ (vector, lane **C**), Xcc∆xopD^*dTALE#1^ (dTALE#1, lane **D**) and Xcc∆xopD^*dTALE#2^ (dTALE#2, lane **E**) infiltrated leaves of the *ProOSCA1.1:OSCA1.1-GFP* transgenic line. (**c**) Ponceau S staining of the membrane used in (**b**). The stained protein bands of Rubisco large subunit (Rubisco-ls) and Rubisco small subunit (Rubisco-ss) were indicated as loading controls. (**b**, **c**) The experiments were repeated twice independently with similar results.

To examine whether or not the virulence function of XopD in planta is solely based on the induction of *OSCA1.1*, we constructed two designer TAL effectors (dTALEs) that transcriptionally activate the *OSCA1.1* promoter (Extended Data Fig. 7a). We transformed the plasmids encoding these dTALEs into the *xopD*-deletion mutant Xcc∆xopD^*^ and infected corresponding transformants (Xcc∆xopD^*dTALE#1^ and Xcc∆xopD^*dTALE#2^) into the leaves of a transgenic line expressing *OSCA1.1-GFP* under control of the *OSCA1.1* promoter (*ProOSCA1.1:OSCA1.1-GFP*). Reverse transcription-quantitative polymerase chain reaction (RT-qPCR) and immunoblotting confirmed elevated levels of *OSCA1.1* transcripts (Fig. 3e) and OSCA1.1-GFP (Extended Data Fig. 7b,c), respectively, indicating dTALE-dependent expressional activation of OSCA1.1 in effector-targeted cells. In addition, microscopic inspection revealed highly induced OSCA1.1-GFP at the plasma membrane of epidermal pavement cells and adjacent guard cells, suggesting that these cells received bacterial effectors (Fig. 3f,g). However, dTALE-induced overexpression of OSCA1.1 did not increase the host susceptibility to Xcc∆xopD^*^ nor to Xcc^*^ (Fig. 3h), and was not sufficient to suppress wilting and necrotic symptoms in Xcc∆xopD^*^ infected leaves (Fig. 3i). This indicates that both transcriptional and translational upregulation of OSCA1.1 activities are necessary but not sufficient for XopD-mediated bacterial pathogenesis.

### XopD promotes *Arabidopsis* abscisic acid signalling

**Fig. 4:**
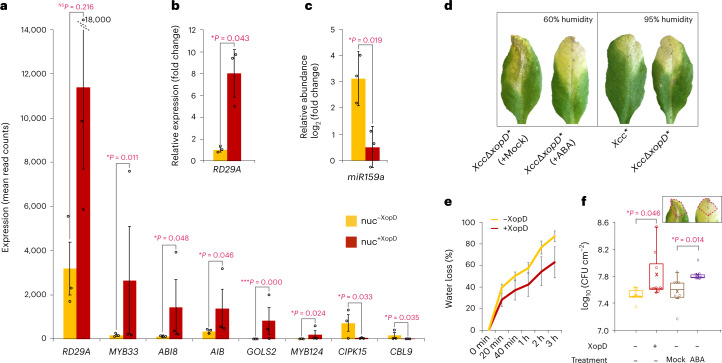
XopD manipulates osmotic signalling to increase the host water level and bacterial growth. **a**, The expression levels (mean read counts) of ABA-responsive genes in nuc^−XopD^ and nuc^+XopD^. Data are presented as mean values ± s.e.m (error bars) from *n* = 3 independent biological replicates. DESeq2 *P* values are from Wald tests corrected for multiple testing using the Benjamini–Hochberg method (nuc^−XopD^ versus nuc^+XopD^; *RD29A*, *P* = 0.216; *MYB33*, *P* = 0.011; *ABI8*, *P* = 0.048; *AIB*, *P* = 0.046; *GOLS2*, *P* = 0.000; *MYB124*, *P* = 0.024; *CIPK15*, *P* = 0.033; *CBL9*, *P* = 0.035). *P < 0.05, ***P < 0.001, NS, not significant (*P* > 0.05). **b**, RT-qPCR analysis of *RD29A* expression in nuc^+XopD^ relative to its expression in nuc^−XopD^. The expression of *TUB2* is used as a control. Data are presented as mean values ± s.d. (error bars) from *n* = 3 independent biological replicates. Statistical significance is determined by a two-sided unpaired *t*-test (*P* = 0.043). *P < 0.05. **c**, Relative abundances of miR159a in nuc^−XopD^ and nuc^+XopD^ compared to its abundance in total nuclei of mock-inoculated leaves. Data are presented as mean values ± s.d. (error bars) from *n* = 3 independent biological replicates and normalized against the abundances of *ACTIN2/8* in each sample. Statistical significance is determined by a two-sided unpaired *t*-test (nuc^−XopD^ versus nuc^+XopD^, *P* = 0.019). *P < 0.05. **d**, Representative leaves infected with indicated bacterial strains under regular (60%) or high (95%) humidity conditions at seven DPI. Mock, treatment with buffer; ABA, treatment with ABA. **e**, The water loss rate of detached Xcc^*^ (+XopD) and Xcc∆xopD^*^ (−XopD) infected leaves at seven DPI. Data are presented as mean values ± s.d. (error bars) from *n* = 8 leaves from four different plants. **f**, A boxplot representing bacterial population density in symptomatic leaf tissues inoculated with Xcc^*^ (+XopD) and Xcc∆xopD^*^ (−XopD) at seven DPI. Horizontal lines from the top show maxima, upper quartile, median, lower quartile and minima values, and cross marks show the mean values. For each treatment, *n* = 8 leaves from four different plants were examined. Statistical significance is determined by a two-sided unpaired *t*-test (no treatment: Xcc∆xopD^*^ inoculated leaves versus Xcc^*^ inoculated leaves, *P* = 0.046; Xcc∆xopD^*^ inoculated leaves: mock treatment versus ABA treatment, *P* = 0.014). *P < 0.05. Small circles (**a**–**c**,**f**) represent data points of individual biological replicates. Source data

What could be the yet unknown host susceptible factor(s) that act in concert with OSCA1.1 in XopD-mediated bacterial virulence? Our eINTACT data also uncovered many DEGs that are involved in regulating *Arabidopsis* abscisic acid (ABA) responses (Fig. 4a). For example, XopD-induced genes included *MYB124* encoding an MYB TF required for stomatal development and ABA-mediated stomatal closure^22^; *AIB* encoding a bHLH TF that activates the transcription of ABA-responsive genes^23^; and *ABI8* and *GOLS2* both encoding positive regulators of ABA responses^24,25^. Notably, the major ABA-responsive gene *RD29A*^26^, had an approximately four-fold increase in transcript levels in nuc^+XopD^ (Fig. 4a), suggesting that XopD enhances ABA response. However, the expressional change of *RD29A* in our RNA sequencing (RNA-seq) data was not statistically significant after correction for multiple testing (Fig. 4a). Since we cannot rule out that our ultra-low-input RNA-seq method, which involves two-step cDNA amplification, might increase variability between biological replicates, we quantified *RD29A* transcripts in nuc^+XopD^ versus nuc^−^^XopD^ by RT-qPCR (Fig. 4b). The RT-qPCR results indeed showed a significant increase of *RD29A* transcripts (Fig. 4b) and confirmed previous results from our RNA-seq analysis (Fig. 4a), thereby demonstrating the reproducibility of the eINTACT method.

We found in nuc^+XopD^ not only increased expression of the positive regulators of ABA responses but reciprocally also decreased expression for several genes encoding negative regulators of ABA signalling, for example, CIPK15 and CBL9, both of which act as Ca^2+^ sensors that negatively modulate ABA sensitivity and biosynthesis^27,28^. Both *cipk15* and *cbl9* mutant plants are hypersensitive to ABA, showing increased expression of ABA-responsive genes and enhanced stomatal closure^27,28^. Similarly, the reduced expression was found in nuc^+XopD^ for the small RNA processors DWA1 and HYL (Table 1) which both play a negative role in the regulation of ABA signalling^29,30^. Exogenous ABA application induces hyperexpression of ABA-responsive genes in *dwa1* mutant plants^30^, and HYL1 represses the ABA-inducible gene *MYB33* via *miR159a*-mediated gene silencing^29,31,32^. To examine the effect of downregulated *HYL1* expression in nuc^+XopD^, we quantified *miR159a* abundance in nuc^−^^XopD^ and nuc^+XopD^ by stem-loop RT-qPCR. We found that the abundance of *miR159a* was markedly (>six-fold) lower in nuc^+XopD^ (Fig. 4c), which coincided with a significant increase in *MYB33* expression in these nuclei (Fig. 4a).

**Extended Data Fig. 8:**
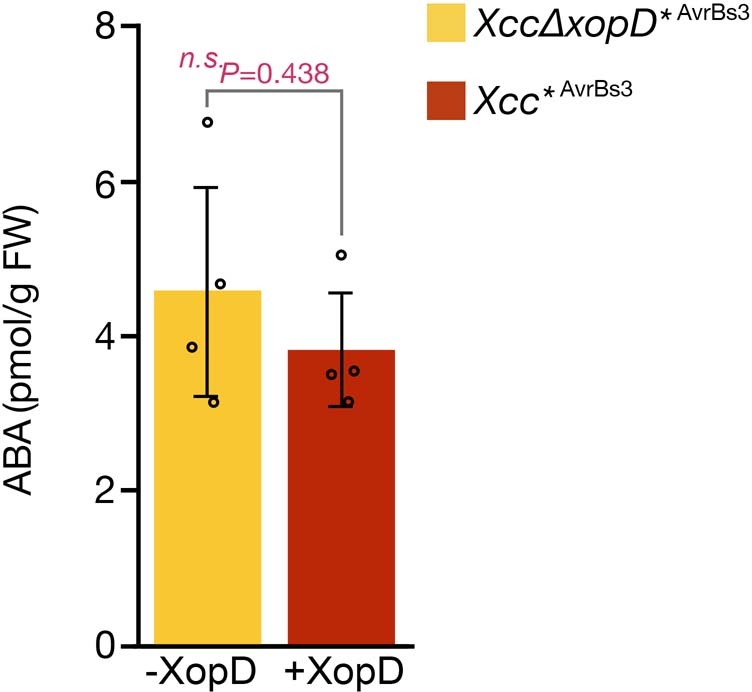
ABA levels in Xcc*^AvrBs3^ and XccΔxopD*^AvrBs3^ infected leaves at five DPI. The ABA contents were quantified by LC–MS for n = 4 biological replicates, with 3 technical repetitions per sample. Data are presented as mean values +/− s.d. (error bars) from n = 4 independent biological replicates. Small circles show data points of individual biological replicates. Statistical significance is determined by two-sided unpaired *t*-test (Xcc*^AvrBs3^ infected leaves versus XccΔxopD*^AvrBs3^ infected leaves, *P* = 0.438). *n.s*., not significant (*P* > 0.05). Source data

The aforementioned data indicate that XopD manipulates the expression of both positive and negative regulators of ABA signalling, being consistent with a working model where XopD induces ABA responses in effector-recipient host cells. Moreover, we did not uncover a significant difference in ABA levels between Xcc*^AvrBs3^ and XccΔxopD*^AvrBs3^ infected leaves by liquid chromatography–mass spectrometry (LC–MS) (Extended Data Fig. 8), suggesting that XopD potentiates ABA signalling responses rather than increasing ABA content.

### Spatio-temporal virulence role of XopD in stomatal closure

It is noteworthy that ABA induces stomatal closure to prevent water loss in response to drought stress^33^. Therefore, manipulation of ABA signalling by XopD could induce stomatal closure resulting in increased water availability in the apoplast to promote bacterial growth. In support of this hypothesis, microscopic studies revealed that epidermal pavement cells and adjacent guard cells that are involved in the regulation of stomatal closure^22^ both received effectors (Figs. 1d–f, 3g). In addition, exogenous ABA application or high humidity (95%) was sufficient to suppress the leaf wilting and early necrosis that is induced by Xcc∆xopD^*^ infection (Fig. 4d). Finally, XopD inhibited water loss in detached Xcc^*^ infected leaves (Fig. 4e) supporting the idea that XopD induces stomatal closure. In this context, it is worth remembering that *OSCA1.1* is crucial for stomatal closure upon osmotic stress^21^ and is necessary for XopD-dependent suppression of leaf necrosis (Fig. 3d). Hence, the wilting and necrotic symptoms and low bacterial infection in *osca1-1* leaves (Fig. 3b–d) were most likely caused by the failure to close stomata, resulting in dehydration of infected tissue that does not support bacterial growth.

**Fig. 5:**
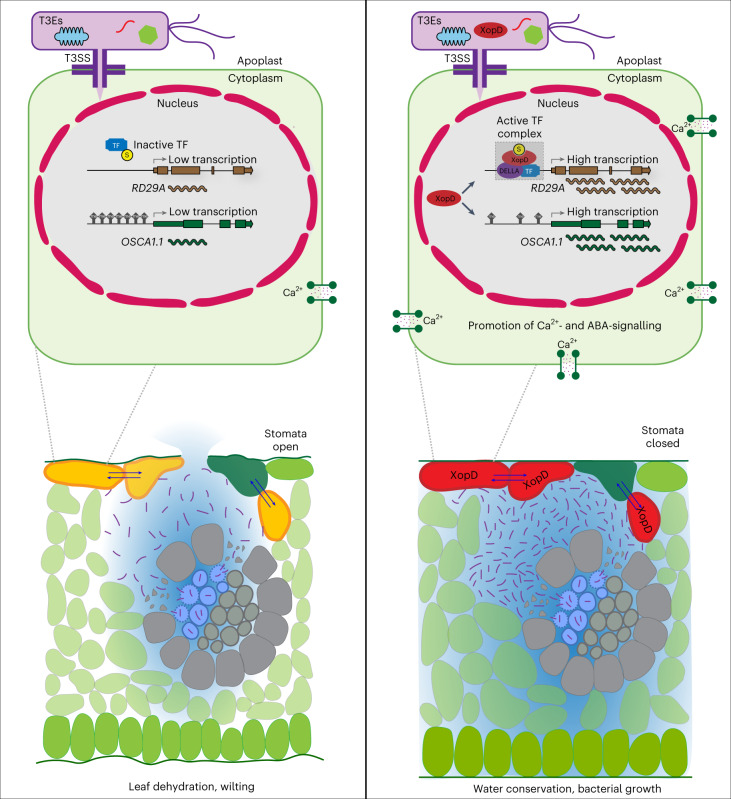
Schematic summary illustrating XopD in planta functions. At the late stage of infection, *Xcc* bacteria (purple rods) break through the plant’s xylem vessels and enter mesophyll apoplast space in symptomatic tissues, where they get access to the cells that are connected to the apoplast and the symplast, such as mesophyll cells, epidermal pavement cells and their neighbouring guard cells, and deliver effectors into these cells. In the XopD recipient cells (red), XopD promotes the expression of ABA-responsive genes (for example, *RD29A*) possibly by controlling functions of DELLA-interacting TFs via its SUMO protease activity, and activates *OSCA1.1* expression correlating with demethylation of the *OSCA1.1* promoter. The XopD-dependent promotion of ABA singling and OSCA1.1-mediated Ca^2+^ increase induces stable stomatal closure for establishing an aqueous apoplastic space that supports bacterial growth. Blue arrows indicate intercellular signals, such as Ca^2+^, miRNAs and hormones. The proposed XopD–DELLA–TF complex is shaded in grey.

**Extended Data Fig. 9:**
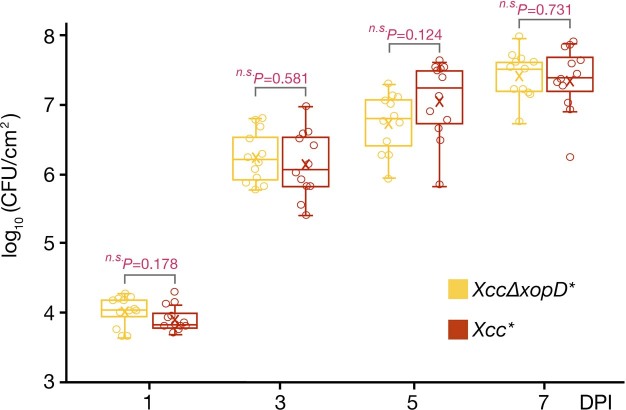
A boxplot representing bacterial population density in *Arabidopsis* leaves inoculated with Xcc∆xopD^*^ or Xcc^*^ at one, three, five and seven DPI. Horizontal lines from the top show maxima, upper quartile, median, lower quartile and minima values; cross marks show the mean values; and small circles show data points of individual biological replicates. The n = 12 leaves were collected from 4–6 different plants. Statistical significance is determined by two-sided unpaired *t*-test (Xcc* infected leaves versus XccΔxopD* infected leaves: 1 DPI, *P* = 0.178; 3 DPI, *P* = 0.581; 5 DPI, *P* = 0.124; 7 DPI, *P* = 0.731). *n.s*., not significant (*P* > 0.05). Source data

After entering leaves via wounds or hydathodes, *Xcc* bacteria rapidly proceed to xylem vessels leading to systemic vascular infections, and eventually adopt a necrotrophic lifestyle. This results in the digestion of the vascular tissues and colonization of the mesophyll apoplast to cause black rot disease^2^. Given that XopD does not promote bacterial growth at the whole-leaf level (Extended Data Fig. 9) but suppresses wilting and necrotic symptoms at the late stages of infection, we hypothesize that XopD induces stomatal closure in a spatio-temporally controlled fashion to increase water levels in the apoplast after *Xcc* has spread out of the plant’s xylem vessels into mesophyll apoplast in symptomatic tissues. The degradation of xylem vessels causes a severe shortage of water in leaves, and accordingly, XopD-mediated stomatal closure inhibits dehydration of the apoplastic space. We propose that the spatio-temporally regulated activity of XopD facilitates prolonged bacterial proliferation during the necrotrophic phase of infection (Figs. 4f,5).

## Discussion

Our study unveils a previously unknown mechanism where the nuclear-targeted *Xanthomonas* effector protein XopD manipulates host OSCA1.1/ABA-mediated stomatal closure to create a humid habitat that favours bacterial proliferation. Particularly, we show that *OSCA1.1* is necessary for the disease-promoting function of XopD in suppressing wilting and early necrosis in infected leaves and a XopD-dependent increase of *OSCA1.1* expression may be required for optimal host susceptibility. The previous study suggested that OSCA1.1 may act upstream of ABA signalling, since stomatal closure induced by exogenous application of ABA was unaffected in the *osca1-1* mutant^21^. However, the downstream components activated by OSCA1.1 in response to osmotic stress remain unknown. Interestingly, recent work on osmotic stress-activated signalling pathways suggests that while Ca^2+^ elevation is not essential in ABA-mediated stomatal closure, the increased Ca^2+^ facilitates faster and steadier level closures^33^. Thus, a possible explanation is that a XopD-dependent increase of *OSCA1.1* transcription might upregulate OSCA1.1 Ca^2+^ channel activities in effector-recipient host cells, resulting in elevated Ca^2+^ levels that accelerate and stabilize ABA-mediated stomatal closure (Fig. 5).

Intriguingly, previous studies on the *Arabidopsis* Ca^2+^-permeable channels OSCA1.3 and OSCA1.7 demonstrated their functional relevance in regulating stomatal closure to prevent *P. syringae* entry^34^. Notably, OSCA1.1 is a hyperosmolality-gated mechanosensitive channel triggered in response to osmotic stress to prevent water loss^21^, and XccΔxopD^*^ infection-induced leaf dehydration potentially provides the hyperosmolality-based gating components for XopD-dependent increase of OSCA1.1 Ca^2+^ channel activity. By contrast, the Ca^2+^-permeable channel activity of OSCA1.3 and OSCA1.7 is activated via phosphorylation by the immune receptor kinase BIK1 upon pathogen perception^34^. This suggests that additional regulatory mechanisms such as post-translational modifications contribute to the regulation of the activities of plant Ca^2+^ channels in response to distinct stimuli. Furthermore, unlike *P. syringae* which is a mesophyll pathogen entering the plant via the stomata^1^, *Xcc* is a vascular pathogen that enters the plant through hydathodes^2^. Therefore, stomatal closure mediated by different Ca^2+^-permeable channels could act as differential hubs of immunity against mesophyll pathogens versus disease promotion of vascular pathogens.

Emphasizing the power of genome-wide studies of transcriptome and epigenome in effector-recipient cells, we uncover 924 XopD-regulated genes involved in diverse mechanisms, for example, gene silencing, negative regulation of gene expression, gametophyte development and tRNA aminoacylation (Fig. 2b and Supplementary Table 2), some of which are correlated with DNA methylation changes in their promoters, for example, *SUVH9* (Fig. 2c). Although we focus on investigating XopD’s function in the transcriptional regulation of osmotic signalling in this study, it would be interesting for future studies to characterize how other previously unknown mechanisms may collectively contribute to the in planta functions of XopD.

Consistent with previous work^10^, we did not observe any notable difference in the transcription of DELLA-dependent GA-responsive genes between nuc^−^^XopD^ and nuc^+XopD^, even though XopD physically interacts with DELLA proteins, which are commonly known to be negative regulators of GA signalling^10^. It is worth noting that in some contexts, DELLA proteins act as positive regulators of ABA signalling^35^. For example, DELLA directly recruits ABI3 and ABI5 TFs to activate ABA-responsive genes during seed germination^35^, while sumoylation of ABI5 attenuates its DNA-binding ability^36^. We found that an ABI5-dependent ABA-responsive gene, *RD29A*^36^, had increased expression in nuc^+XopD^ in our transcriptome analysis (Fig. 4a,b), making it plausible that XopD interacts with DELLA to manipulate functions of DELLA-interacting TFs via its SUMO protease activity to activate ABA responses (Fig. 5). In this context, a XopD homologue, XopD^*Xe*^ from the *X.* *euvesicatoria* strain 85-10 (*Xe*85-10), has been shown to deSUMOylate and destabilize the tomato ethylene-responsive transcription factor SlERF4 to suppress ethylene-mediated defence responses^37^. Protein structure analysis shows that XopD^*Xe*^ contains an N-terminal extension sequence and a putative DNA-binding helix-loop-helix domain which are absent in XopD^9^ (Extended Data Fig. 1). Moreover, XopD is unable to complement the *Xe*85-10ΔxopD^*Xe*^ mutant^9^, suggesting distinct functions of XopD and XopD^*Xe*^.

In closing, we have established a new concept to recover nuclei of effector-recipient host cells from infected leaf tissues and demonstrated that the eINTACT system enables us to uncover effector-dependent host transcriptional and epigenetic changes that were undetectable when bulk-infected leaf tissues were used as study materials. In addition, eINTACT is uniquely suited for unveiling in planta functions of effectors that are subjected to dose-dependent, spatio-temporal and cell-type-specific guidelines. Currently, single-cell RNA-seq is increasingly used to measure gene expression levels in individual cell types in plants^38^. However, bacterial infection induces changes in cell turgor, cell wall integrity and cell sizes that inevitably affect cell isolation and sorting processes, restricting the usage of single-cell RNA-seq in plant pathology studies^38^. Thus, eINTACT stands as a unique, low-cost and convenient method for isolating high-quality nuclei in bacterial effector-targeted host cells for subsequent transcriptomic and epigenomic analyses without specialized and expensive equipment. In this study, we used *Xcc*-mediated delivery of AvrBs3 to activate the eINTACT reporter in *Arabidopsis* cells for studying the *Xcc* effector XopD, it is also feasible to transfer AvrBs3 to other bacterial species, for example, the widely used model pathogen *P. syringae*. Therefore, we anticipate that the newly established eINTACT system will provide a basis to accurately elucidate in planta function of effectors from numerous gram-negative plant bacteria in native infection contexts.

## Methods

Sequences of oligonucleotides used in this work are listed in Supplementary Table 6. Plasmids and bacterial strains used in this work are summarized in Supplementary Table 7. All constructs were verified by Sanger sequencing.

### Plant growth conditions

*Arabidopsis thaliana* accession Col-0 was used as the wild-type plant in this work. The homozygous seeds of *ProXVE:XopD* transgenic line no. 38 expressing XopD under control of a β-estradiol-inducible promoter, *osca1-1* mutant, *ProOSCA1.1:OSCA1.1-GFP* and *Pro35S:OSCA1.1/osca1-1* lines were established in the previous studies^11,21^.

Before sowing, seeds were stratified in 0.1% agar at 4 °C for three days. For generating the transgenic line and for the β-estradiol treatment, plants were grown on soil or agar plates containing 1/2 Murashige and Skoog medium (MS), 0.8% agar and the appropriate antibiotics in growth chambers under long-day conditions (16 h of light and 8 h of dark) at 22 °C with relative humidity 50%.

For infection assays, plants were grown on soil in growth chambers under short-day conditions (8 h of light and 16 h of dark) at 22 °C with relative humidity 50% for five weeks. Two days before infections, plants were transferred to a short-day growth chamber at 25 °C with relative humidity 60%, which is the upper temperature and humidity limits for optimal *Arabidopsis* growth^39^. The warmer and higher relative humidity is favourable for *Xcc* infection and promotes earlier symptom development by two days. The infected plants were kept in the 25 °C short-day chamber with relative humidity 60% or 95%. The light condition was constructed by a mixture of Cool White and Gro-Lux Wide Spectrum fluorescent lights, with a fluence rate of 125 to 175 μmol m^−2 ^s^−1^.

### Generation of the eINTACT reporter *Arabidopsis* lines

For the preparation of the *ProBs3*:*RedNTF:tNOS* construct (designated pYY1704), a Gateway-compatible entry plasmid carrying the 344-base pair (bp) promoter sequence of pepper *Bs3* gene^18^ was cloned into a pGREEN-IIS-based destination vector containing the attR1-attR2 Gateway recombination cassette (pFK-386). Gateway LR clonase II enzyme mix (Invitrogen) was used for Gateway reactions. Subsequently, a nopaline synthase terminator was cloned after the attR1-attR2 cassette by sticky end cloning using restriction enzymes *Eco*RI and *Bam*HI. Finally, the DNA fragment of the NTF protein from plasmid pYY1204^16^, was placed between the *Bs3* promoter and the nopaline synthase terminator by sticky end cloning using restriction enzyme *Eco*RI.

The *Pro35S*:*RedNTF:rbcs* construct (designated pYY1705) was generated via Gateway recombination of the entry plasmid pYY1204^16^ and a pGREEN-IIS-based destination vector harbouring the *35S* promoter in the front of the attR1-attR2 Gateway recombination cassette (pFK-209).

For generating the eINTACT reporter line and the *Pro35S*-INTACT reporter lines, pYY1704 and pYY1705 were transformed into homozygous *ProUBQ10*:*BirA* lines^16^, making use of *Agrobacterium tumefaciens* strain *ASE* and the floral dip method^40^. Homozygous lines were selected on 1/2 MS agar plates containing 50 μg ml^−1^ of kanamycin.

### Construction of dTALEs

The two 18-bp TAL effector binding elements (EBEs), preceded by thymine (T), were identified approximately 50 bp upstream of the *OSCA1.1* transcription start site (TSS). Their nucleotide sequences are: EBE#1, **T**CTTGTGTGTTTCTCGCGT; EBE#2, **T**ACTTCATTCATCACTGCT. To clone the dTALEs targeting these EBEs, a DNA fragment encoding AvrBs3 N-terminal (290 aa) and C-terminal region (281 aa), flanked with *Bsa*I restriction enzyme sites, was amplified by PCR and subsequently cloned into a pENTR CACC-AAGG plasmid vector^41^, yielding pENTR TALE N/C. Subsequently, the TALE-*Bam*HI fragment in the pSKX1-ArtTAL vector^42^ was replaced by the AvrBs3 N/C fragment from pENTR TALE N/C making use of *Bam*HI restriction enzyme, yielding pSKX1-TALE N/C. The repeat variable diresidues of dTALEs targeting the two EBEs, dTALE#1, HD-NG-NG-NH-NG-NH-NG-NH-NG-NG-NG-HD-NG-HD-NH-HD-NH-NG; dTALE#2, NI-HD-NG-NG-HD-NI-NG-NG-HD-NI-NG-HD-NI-HD-NG-NH-HD-NG, were created by modular assembly and finally cloned into pSKX1-TALE N/C making use of *Bpi*I restriction enzyme^43^, yielding pSKX1-dTALE#1 and pSKX1-dTALE#2.

### *Xcc* strains and growth conditions

To create 8004∆*avrAC∆xopAM∆xopD* (Xcc∆xopD^*^) mutant strain, the *xopD* deletion was introduced in *Xcc* double deletion mutant 8004∆*avrAC∆xopAM* (Xcc^*^) using the SacB system with a modified pK18 suicide vector^19^ and verified by PCR. To create Xcc^*AvrBs3^ (8004∆*avrAC∆xopAM pDS300F*) and Xcc∆xopD^*AvrBs3^ (8004∆*avrAC∆xopAM∆xopD pDS300F*), a pDSK-based plasmid expressing AvrBs3 (pDS300F)^18^ was conjugated into the double and triple mutants, respectively, by triparental mating^19^. Xcc^*vec1^ and Xcc^*vec2^ were prepared by transforming Xcc^*^ with an empty pDSK and pSKX1 plasmid, by electroporation. To generate bacterial strains that deliver dTALEs, Xcc^*^ or Xcc∆xopD^*^ were transformed with pSKX1-dTALE#1 and pSKX1-dTALE#2 by electroporation, yielding Xcc^*dTALE#1^, Xcc^*dTALE#2^, Xcc∆xopD^*dTALE#1^, Xcc∆xopD^*dTALE#2^. All *Xcc* strains were grown on nutrient yeast glycerol agar plates at 28 °C, and antibiotic selection was carried out by using the following concentrations (in µg ml^−1^): rifampicin, 50; spectinomycin, 40; gentamycin, 15.

### Infection assay

When the wounding inoculation method was used, fully expanded leaves of five-week-old plants were inoculated with bacterial inoculum (10^8 ^CFU ml^−1^, OD: 0.1 in 1 mM MgCl_2_), by piercing the central leaf vein three times with a needle that had been dipped in bacterial inoculum^19^. Plants inoculated with 1 mM MgCl_2_ were used as mock-inoculated control. When the infiltration method was used, the leaf abaxial sides were infiltrated with bacterial inoculum using a blunt-end syringe.

After inoculation, the trays of plants were covered with clear plastic covers and sealed to keep nearly 100% relative humidity for 24 h. At one DPI, the covers were removed to keep the infected plants with a relative humidity of 60%. To keep the infected plants with high relative humidity (95%) in some experiments, the covers were slightly opened. A mobile humidity meter was used to monitor the humidity in the trays.

### Microscopy

A LEICA DMI3000 B imaging system was used for all imaging in this work, using bright field, 4′,6-diamidino-2-phenylindole (DAPI), mCherry and GFP filters.

### Sample collection and purification of effector-recipient nuclei

Three sets of leaf samples were harvested and processed independently of each other. For each set, approximately 650 leaves of eINTACT reporter plants were inoculated with Xcc^*AvrBs3^ or Xcc ∆xopD^*AvrBs3^, using the wounding method. The inoculated leaves were collected at five DPI, and immediately frozen in 50 ml falcon tubes suspended in liquid nitrogen. The samples were stored at −80 °C before INTACT purification. The INTACT experiments were performed as described previously^16,17^. The purity and yield of nuclei were assessed by microscopy. From each leaf sample, ∼2.5 × 10^5^ nuclei were yielded and divided into 1 × 10^5^ for preparing an RNA-seq library, 1 × 10^5^ for Stem-loop qPCR analysis of miRNAs, and 5 × 10^4^ for preparing an enzymatic methyl-sequencing library. Nuclear samples were stored at −80 °C before further experiments.

### Western blotting

To validate the eINTACT system, total nuclei from leaves of eINTACT reporter line infected with Xcc^*vec1^ or Xcc^*AvrBs3^ were prepared following the protocol of preparing for the input nuclei before INTACT purification^16,17^. Effector-recipient nuclei were purified from Xcc^*AvrBs3^*-*infected leaves by INTACT. The total nuclei from leaves of the *Pro35S*-INTACT reporter line were used as a control. Nuclear protein samples were prepared by mixing total nuclei and ∼10,000 INTACT-purified nuclei with 1× SDS buffer, denatured by boiling at 95 °C for 5 min and analysed by Western blotting. The biotinylated-NTF protein (∼42 kDa) was detected using streptavidin alkaline phosphatase (Promega), and signals were developed by a colour reaction using NBT-BCIP solution (Roche). The amount of H3 protein (∼15 kDa) was measured as an internal control of the number of nuclei in each sample, using an anti-H3 antibody (Millipore, catalogue no. 17-10254) at 1:1,000 dilution and an anti-rabbit secondary antibody (IRdye680/LI-COR, catalogue no. 925-68073) at 1:10,000 dilution. The conjugated fluorophore signal was visualized with an Amersham Typhoon scanner (GE Healthcare Life Sciences) using a BPFR 700 filter at 680 nm.

To examine the OSCA1.1-GFP overexpression induced by bacterial-delivered dTALEs, the bacterial infiltrated leaves of the *ProOSCA1.1:OSCA1.1-GFP* transgenic line were collected at one DPI and flash-frozen in liquid nitrogen. Leaf samples were ground and mixed with 2× SDS buffer, denatured by boiling at 95 °C for 5 min and analysed by Western blotting. A mouse monoclonal, HRP-conjugated anti-GFP antibody (Santa Cruz Biotechnology, catalogue no. sc-9996 HRP) was used at 1:2,000 dilution to detected OSCA1.1-GFP. The signals were developed by Clarity Western ECL Substrate (Bio-Rad) and detected in an Amersham Imager 600. After the detection of GFP signals, the western blotting membrane was stained in Ponceau S and washed with water for visualization of all protein bands in the samples. The stained protein bands of Rubisco subunits were used as loading controls.

Sizes of the detected proteins were judged according to a PageRuler Prestained Protein Ladder (Thermo Fisher Scientific).

### RNA sequencing

RNA-seq was performed in three independent biological replicates for nuc^+XopD^ and nuc^−^^XopD^. For each sample, approximately 500 pg nuclear RNA was extracted from ∼10^5^ nuclei using the RNeasy Plus Micro Kit (Qiagen) and further treated with DNase I (0.05 U per μl, Thermo Fisher Scientific) for 30 min at 37 °C to remove any contaminating genomic DNA. The double-stranded (ds) cDNA was synthesized from ∼500 pg RNA by two rounds of linear amplification using the SMARTer Ultra Low Input RNA for Illumina Sequencing-HV kit (Clontech) according to the manufacturer’s instructions. The concentration and yield of the amplified cDNA was determined using the Qubit dsDNA High Sensitivity Assay Kit (Invitrogen). RNA-seq libraries were prepared using the Low Input Library Prep Kit (Clontech) according to the manufacturer’s instructions. The quality and quantity of the RNA-seq libraries was examined using the High Sensitivity DNA Kit (Agilent). Sequencing was performed on NovaSeq 6000 system (Novogene, UK). From 33.2 to 40.9 millions of 2× 150-bp paired-end reads that passed the Illumina quality control filter were collected for each sample (Supplementary Table 8).

### Transcriptome data analysis

Adapter removal and quality trimming of reads were done using AdapterRemoval v.2.1.7 (parameters: -min quality 20, -min length 50)^44^. RNA-seq reads were mapped to the *Arabidopsis* reference transcriptome TAIR10 v.47, with ribosomal RNA regions (2:3471-9557; 3:14197350-14203988) masked, using TopHat 2.0.13 (no-mixed alignments; up to 20 secondary alignments; no new junctions)^45^. Read counts covering transcripts were computed using the featureCounts program in R^46^, and submitted to differential gene expression analysis in DESeq2 (v.1.32.0) in R (v.4.1.0) (default parameters; significance conditions: base expression > 5, false discovery rate (FDR) adjusted *P* value < 0.05, |log_2_FC| > 1)^47^.

### Gene ontology term analysis

The online tool AmiGO 2^48^ (PANTHER overrepresentation test released 20210224; GO Ontology database released 2 July 2021) was used to identify GO terms in biological processes that are over or under-represented in XopD-dependent DEGs. Binomial tests were performed and FDR adjusted *P* value < 0.05 was considered as significant. Gene-concept network plot was conducted by the cnetplot function in the clusterProfiler package (clusterProfiler v.3.18.1)^49^ in R (v.4.0.5).

### Enzymatic methyl-sequencing

Approximately 5 ng of genomic DNA was extracted from ∼1.5 × 10^5^ nuclei of each type of sample, using DNeasy Plant Pro Kit (QIAGEN), and treated with RNase A (Thermo Scientific) to remove any contaminating RNA. Subsequently, ∼5 ng DNA (including 0.02 ng of unmethylated lambda DNA and 0.001 ng of >96% methylated pUC19 DNA spiked in) was sheared to 100–500-bp fragments using a focused ultrasonicator (Covaris E220 system), in microtubes at a setting of 175 peak incident power, 10 dc, 200 cpb for 40 s. Enzymatic methyl-sequencing (EM-seq) libraries were prepared from sheared DNA using NEBNext Enzymatic Methyl-seq Kit following the manufacturer instructions (New England BioLabs). Due to the amount of starting DNA material being lower than 10 ng, the minimum recommended amount of starting material by the manufacturer, we added two PCR cycles at the final step of PCR amplification of the sequencing libraries. Libraries were sequenced on NovaSeq 6000 system (Novogene) for collecting 2× 150-bp paired-end reads that passed the Illumina quality control filter (Supplementary Table 8).

### DNA methylation data analysis

EM-seq reads were adapter- and quality-trimmed by trim_galore v.0.6.4^50^, before mapping to the *Arabidopsis* reference genome (TAIR10 v.47) and control sequences (lambda and pUC19 DNA), using Bowtie v.2.2.3 (score–min L, 0, −0.6)^51^. Deduplication and methylation inference were done in Bismark v.0.22.3^52^, with an additional 3 bp of read ends ignored based on M-bias. Final cytosine counts at a minimum coverage of four reads were tested for false methylation, per context, by fitting a binomial model based on the negative control (unmethylated lambda DNA). Benjamini–Hochberg procedure was applied with the threshold FDR of 5%.

Given the limited numbers of effector-targeted nuclei available from eINTACT, we combined ∼5 × 10^4^ nuclei from each of the three biological replicates (Sample collection and purification of effector-recipient nuclei) to make a pooled sample of each type of nuclei. Therefore, to identify differentially methylated regions (DMRs) for each context (CG, CHG, CHH), we used DSS-single (v.2.38) in R (v.4.0.3), a statistical method using information from neighbouring CG sites for estimate biological variation in a single sample, which has been proven to have greater sensitivity and accuracy to yield the most biologically meaningful results even without replicates^53^. For DMR calling, per residue *P* value threshold was set to 0.05. Furthermore, the obtained DMR list was filtered based on stringent cut-offs of length >100 bp, mean methylation difference >0.15, test statistic areaStat >10. A locally installed WashU Epigenome Browser was used for visualizing DNA methylation at single-base resolution^54^.

The DMR-overlapping genomic features were extracted using the *Arabidopsis* reference genome TAIR10 v.47 and the Araport11 annotation files for transposon and pseudogene locations. To associate DMRs with protein-coding genes, DMRs located within 3-kb proximal regions, 3 kb upstream of the TSS to 3 kb downstream of the transcription termination site, were extracted. The relative positions to genes were calculated from the middle of DMR to closest gene termini (TSS or transcription termination site).

### RT-qPCR

To verify the expression changes of *RD29A* in nuc^+XopD^ versus nuc^−^^XopD^ by RT-qPCR, ∼100 pg amplified ds cDNA prepared from nuclear RNA samples (RNA sequencing) was used in qPCR reactions. To examine the relative gene expression in leave samples, RNA was extracted from one to two leaves from each sample type using the RNeasy Plant kit (Qiagen), and treated with DNase I (Thermo Fisher Scientific) to remove any contaminating genomic DNA. cDNA synthesis was performed using the RevertAid First Strand cDNA synthesis kit (Thermo Fisher Scientific) and the oligo-dT primer. PCR reaction was performed with gene-specific primers and HS Taq Master Mix (Biozyme).

The cDNA samples were used as the templates for qPCR reactions, using PowerUP SYBR Green Master Mix (Applied Biosystems) and gene-specific primers, in a CFX384 Touch Real-Time PCR machine (Bio-Rad). The qPCR cycle threshold (Ct) values of each examined genes were normalized to the average Ct values of *TUB2* house-keeping gene, and the relative gene expression was calculated using the 2^−ΔΔCt^ method. Because amplification signals from the XopD samples in DMSO-treated *ProXVE:XopD* transgenic plants did not cross the detection threshold, the Ct value was set to 40, the allotted number of cycles used in the PCR programme, to be able to conservatively estimate the differences in expression.

The qPCR analysis was performed using two or three independent biological replicates of each sample type that were grown at the same time but harvested and processed independently of each other with three technical replications per sample. Statistical differences were calculated by two-sided unpaired *t*-test analysis.

### Stem-loop qPCR analysis of miRNAs

The nuc^+XopD^ and nuc^−^^XopD^ samples were used with total nuclei from mock-inoculated plants used as a control. Expression levels of miR159a in these samples were measured by stem-loop qPCR following the protocol in previous works^31^ with some modifications. Total RNA was prepared from ∼10^5^ nuclei from each nucleus sample, using TRIzol reagent (Invitrogen) following the manufacture’s instructions, and subsequently treated with DNase I to remove any contaminating genomic DNA. cDNA synthesis was performed using PromeScript Reverse Transcriptase (Takara) with a mixture of stem-loop RT primers for each miRNA and oligo-dT primer for house-keeping genes *ACTIN2/8*. Before qPCR, the cDNA for each of the biological replicates was pre-amplified for 12 cycles using Phusion Polymerase (Thermo Scientific) and the miRNA specific primers. The PCR condition was one cycle at 95 °C for 5 min and 12 cycles of 96 °C for 10 s followed by 60 °C for 30 s. The pre-amplified products were precipitated with the addition of glycogen (Thermo Scientific), and dissolved in 10 μl of DEPC water. The entire sample was then used as the template for the qPCR reaction performed in a 20 μl reaction volume in a Bio-Rad CFX connect apparatus using the SYBR Green Master Mix (Bio-Rad) for 40 cycles. The Ct values of each miRNA were normalized to the average Ct values of *ACTIN2/8*, and fold change was calculated by the 2^−ΔΔCt^ method. qPCR analysis was performed for three independent biological replicates of each sample type with two technical replications per sample. Statistical differences were calculated by two-sided unpaired *t*-test analysis and corrected with FDR for multiple pair comparisons.

### Exogenous application of ABA

The plants were inoculated with relative bacterial strains by the wounding method, as described in the Infection assay section. Every day starting from one DPI, 20 μM ABA (Duchefa) in 100 μM NaOH and 0.02% Silwet L–77 was sprayed onto Xcc∆xopD^*^-infected leaves. Leaves sprayed with 100 μM NaOH and 0.02% Silwet L–77 were used as the mock control.

### β-estradiol treatment

The *ProXVE:XopD* seedlings (transgenic line no. 38)^11^ were germinated and grown on 1/2 MS agar plates containing 12.5 µg ml^−1^ of Hygromycin, in growth chambers under long-day conditions. The two-week-old seedlings were transferred into 1/2 MS liquid medium containing 20 μM β-estradiol (dissolved in DMSO) or DMSO only, and incubated on a 60 rpm shaker placed in the long-day growth chamber. After a 48 h incubation, the seedlings were collected and flash-frozen in liquid nitrogen, before analysing the expression of the genes of interest by RT-qPCR.

### Measurement of ABA content

The Xcc*^AvrBs3^ and XccΔxopD*^AvrBs3^ inoculated leaves of the eINTACT reporter line were collected at five DPI. The leaf tissues were weighed (50 mg ± 10%) and transferred into separate 2 ml safe-lock tubes containing a 5 mm steel ball. The sample tubes were fresh-frozen in liquid nitrogen and stored at −80 °C. For extracting endogenous ABA, the leaf tissue was ground using a Retch mill (2× 15 s) with intermittent cooling in liquid nitrogen. All solvents (LC–MS grade) used in the following steps were prechilled at 8 °C. Two hundred μl 80% MeOH containing 50 nM D6-ABA (isotopic standard, OlChemIm) were added to the ground sample. The mixture was incubated for 5 min in an ultrasonic bath at 16 °C, and subsequently centrifuged at 4 °C, 18620 RCF for 5 min. The supernatant was transferred to a prechilled tube. The remaining pellet was re-extracted with 600 µl H_2_O 0.1% formic acid using the same conditions as in the previous step. The combined supernatant fractions were directly measured using targeted LC–MS. The LC–MS analysis was performed as described previously^55^ except for using a Luna Omega Polar C18 column (3 μm; 100 Å; 150 × 0.5 mm; Phenomenex), a Luna C18(2) trap column (5 μm; 100 Å; 20 × 0.5 mm; Phenomenex) with an increased column temperature of 55 °C and a flow rate of 28 µl min^−1^ for the main column. The ABA content in a sample was normalized against the D6-ABA values.

LC–MS analysis was performed for four independent biological replicates of each sample type that were grown and infected at different time, with three technical replications per sample. Statistical differences were calculated by two-sided unpaired *t*-test analysis.

### Measurement of water loss rate

Eight leaves infected with either Xcc^*^ or Xcc∆xopD^*^, from four different plants kept with relative humidity 95%, were excised at seven DPI and placed at room condition (approximately 25 °C with relative humidity 35%). The leaves were weighed at time points of 0, 20, 40, 60, 120 and 180 min. The water loss rate was calculated as percentage of initial fresh weight. The mean values for eight leaves were calculated. The experiment was repeated twice with similar results.

### Determination of in planta bacterial populations

Whole leaves or symptomatic leaf areas infected with a given *Xcc* strain were collected at seven DPI and placed between clear plastic foils. The leaf sizes (cm^2^) were determined on scanned images using ImageJ software (v.2.0.0-rc-69/1.52p). Each leaf sample was then homogenized in 400 µl of sterile water in safe-lock tubes containing a 5 mm ceramic ball using the TissueLyser II (QIAGEN). Serial dilutions of the homogenates were performed, and a 4 µl drop of each dilution was spotted three times on nutrient yeast glycerol plates supplemented with appropriate antibiotics. The plates were incubated at 28 °C for 48 h, and colonies were counted in spots containing 3 to 30 colonies. The mean of bacterial populations per cm^2^ of leaf tissue was calculated with three technical replications per sample. Statistical differences (*P* < 0.05) between two sample types were calculated by two-sided unpaired *t*-test analysis, and among all (>2) sample types were analysed by ANOVA followed by post hoc Tukey’s honestly significant difference. Experiments were performed at least twice with similar results.

### Reporting summary

Further information on research design is available in the Nature Portfolio Reporting Summary linked to this article.

## Supplementary information

Supplementary information Supplementary Text.

Reporting Summary

Supplementary Tables Supplementary Tables 1–8. Supplementary Table 1: Differential gene expression between nuc^+XopD^ and nuc^-XopD^. Supplementary Table 2: GO enrichment in biological process of significantly differentially expressed genes. Supplementary Table 3: Profiles of all CG-, CHG- and CHH- DMRs comparing nuc^+XopD^ vs nuc^-XopD^. Supplementary Table 4: The 19 DEGs with DMRs located within 3-kb proximal promoter regions. Supplementary Table 5: Expression changes of selected SA-related defence genes in nuc^+XopD^ vs nuc^-XopD^. Supplementary Table 6: Sequences of primers. Supplementary Table 7: Plasmids and *Xcc* strains used in this study. Supplementary Table 8: RNA-seq and Methyl-seq data characteristics.

## Data Availability

RNA-seq and EM-seq data have been deposited with ArrayExpress database (https://www.ebi.ac.uk/biostudies/arrayexpress) accession numbers E-MTAB-10280 and E-MTAB-10281. The *Arabidopsis* reference genome (TAIR10 v.47) and gene annotation are publicly available at http://ftp.ensemblgenomes.org/pub/plants/release-47/. Araport11 annotation for transposons and pseudogenes are publicly available at https://datacommons.cyverse.org/browse/iplant/home/araport/public_data/Araport11_Release_201606/annotation. The authors declare that all other data supporting the findings of this study are available in the main text or the supplementary materials. Source data are provided with this paper.

## Source data

Source Data Fig. 2 Statistical Source Data.
Source Data Fig. 3 Statistical Source Data.
Source Data Fig. 4 Statistical Source Data.
Source Data Extended Data Fig. 2 Statistical Source Data.
Source Data Extended Data Fig. 4 Statistical Source Data.
Source Data Extended Data Fig. 6 Statistical Source Data.
Source Data Extended Data Fig. 8 Statistical Source Data.
Source Data Extended Data Fig. 9 Statistical Source Data.
