# Supplementary material for: The eINTACT system dissects bacterial exploitation of plant osmosignalling to enhance virulence
Source: Nat Plants. 2022 Dec 22;9(1):128–41. doi: 10.1038/s41477-022-01302-y (PMC9873569; doi:10.1038/s41477-022-01302-y)
Supplement: Supplementary file 1 — Supplementary Text. [file 41477_2022_1302_MOESM1_ESM.pdf]

nature plants

Article https://doi.org/10.1038/s41477-022-01302-y

The eINTACT system dissects bacterial exploitation of plant osmosignalling to enhance virulence

In the format provided by the authors and unedited

SUPPLEMENTARY INFORMATION

This PDF file includes:

- **Supplementary Text**
- **Titles of Supplementary Tables 1-8**

Supplementary Text. A detailed description of the *Arabidopsis*-*Xcc* eINTACT system (Fig. 1a, b). (**a**) The bacterial mutant strain *Xcc*^{*AvrBs3} delivers the TAL effector AvrBs3 together with other type III effectors (T3Es) to targeted host cells via the unique type III secretion system (T3SS) during infection. The *Arabidopsis* eINTACT reporter line expresses two transgenes: the red fluorescent nuclear envelope-targeting protein (NTF), containing the WPP domain of the *Arabidopsis* RAN GTPASE ACTIVATING PROTEIN 1 (RanGAP1; *At3g63130*) for nuclear envelope targeting, red fluorescent protein (mCherry) to allow visualization, and the biotin ligase recognition peptide (BLRP), is expressed under the control of pepper *Bs3* promoter (*LOC107857984*), and *E. coli* biotin ligase (BirA) is constitutively expressed under the control of *Arabidopsis UBQ10* gene (*At4g05320*) promoter. AvrBs3 induces the expression of NTF only in effector-targeted host cells, leading to biotinylation (**B**) of the nuclear envelope. T3Es, for example, *Xanthomonas* outer protein D (XopD), are indicated with colour icons. (**b**) Making use of the biochemical interaction between biotin and streptavidin, the biotin-tagged nuclei can be isolated using streptavidin (S)-coated magnetic beads by affinity-based purification (INTACT method).

Titles of Supplementary Tables 1-8

Supplementary Table 1. Differential gene expression between nuc^{+XopD} and nuc^{-XopD}.

Supplementary Table 2. GO enrichment in biological process of significantly differentially-expressed genes.

Supplementary Table 3. Profiles of all CG-, CHG- and CHH- DMRs comparing nuc^{+XopD} vs nuc^{-XopD}.

Supplementary Table 4. The 19 DEGs with DMRs located within 3-kb proximal promoter regions.

Supplementary Table 5. Expression changes of selected SA-related defense genes in nuc^{+XopD} vs nuc^{-XopD}.

Supplementary Table 6. Sequences of primers.

Supplementary Table 7. Plasmids and *Xcc* strains used in this study.

Supplementary Table 8. RNA-seq and Methyl-seq data characteristics.
