# Supplementary material for: The eINTACT system dissects bacterial exploitation of plant osmosignalling to enhance virulence
Source: Nat Plants. 2022 Dec 22;9(1):128–41. doi: 10.1038/s41477-022-01302-y (PMC9873569; doi:10.1038/s41477-022-01302-y)
Supplement: Supplementary file 2 — Reporting Summary [file 41477_2022_1302_MOESM2_ESM.pdf]

nature portfolio

Corresponding author(s): Yuan You

Last updated by author(s): Oct 22, 2022

Reporting Summary

Nature Portfolio wishes to improve the reproducibility of the work that we publish. This form provides structure for consistency and transparency in reporting. For further information on Nature Portfolio policies, see our Editorial Policies and the Editorial Policy Checklist.

Statistics

For all statistical analyses, confirm that the following items are present in the figure legend, table legend, main text, or Methods section.

n/a	Confirmed	
☐	☒	The exact sample size (*n*) for each experimental group/condition, given as a discrete number and unit of measurement
☐	☒	A statement on whether measurements were taken from distinct samples or whether the same sample was measured repeatedly
☐	☒	The statistical test(s) used AND whether they are one- or two-sided<br>*Only common tests should be described solely by name; describe more complex techniques in the Methods section.*
☐	☒	A description of all covariates tested
☐	☒	A description of any assumptions or corrections, such as tests of normality and adjustment for multiple comparisons
☐	☒	A full description of the statistical parameters including central tendency (e.g. means) or other basic estimates (e.g. regression coefficient) AND variation (e.g. standard deviation) or associated estimates of uncertainty (e.g. confidence intervals)
☐	☒	For null hypothesis testing, the test statistic (e.g. *F*, *t*, *r*) with confidence intervals, effect sizes, degrees of freedom and *P* value noted<br>*Give P values as exact values whenever suitable.*
☒	☐	For Bayesian analysis, information on the choice of priors and Markov chain Monte Carlo settings
☐	☒	For hierarchical and complex designs, identification of the appropriate level for tests and full reporting of outcomes
☐	☒	Estimates of effect sizes (e.g. Cohen's *d*, Pearson's *r*), indicating how they were calculated

Our web collection on statistics for biologists contains articles on many of the points above.

Software and code

Policy information about availability of computer code

Data collection	Illumina sequencing reads were collected on NovaSeq 6000 system (Novogene, UK) and passed the Illumina quality control filter.<br>The Arabidopsis reference genome (TAIR10 version 47) and gene annotation are publicly available at http://ftp.ensemblgenomes.org/pub/plants/release-47/. Araport11 annotation for transposons and pseudogenes are publicly available at https://datacommons.cyverse.org/browse/iplant/home/araport/public_data/Araport11_Release_201606/annotation. Microscopy images were taken by a LEICA DMI3000 B imaging system. Leaf sizes were determined on scanned images using ImageJ software (version 2.0.0-rc-69/1.52p).
Data analysis	All software and code used in data analysis are publicly available, open source and their running parameters are disclosed in the Methods.<br>>>Transcriptome Data Analysis: adapter removal and quality trimming using AdapterRemoval version 2.1.7 (Schubert et al., BMC Res Notes, 2016), mapping using TopHat 2.0.13 (Trapnell et al., Bioinformatics, 2009), counting reads using the featureCounts program in R (Liao et al., Bioinformatics, 2014), differential gene expression analysis using DESeq2 (version 1.32.0) in R (version 4.1.0) (Love et al., Genome Biol, 2014).<br>>>DNA Methylation Data Analysis: adapter removal and quality trimming using trim_galore 0.6.4 (Martin, doi:https://doi.org/10.14806/ej.17.1.200), mapping using Bowtie 2.2.3 (Langmead et al., Nat Methods, 2012), deduplication and methylation inference using Bismark v0.22.3 (Krueger et al., Bioinformatics, 2011), identifying differentially methylated regions using DSS-single (version 2.38) in R (version 4.0.3) (Wu et al., Nucleic Acids Res, 2015) and visualizing DNA methylation using WashU Epigenome Browser (Zhou et al., Nat Methods, 2011)<br>>>Image analysis: ImageJ software (version 2.0.0-rc-69/1.52p).<br>>>GO analysis: The online tool AmiGO 2 (http://amigo.geneontology.org/amigo; PANTHER overrepresentation test released 20210224; GO Ontology database released 2021-07-02).<br>>>The gene-concept network: The cnetplot function in the clusterProfiler package (clusterProfiler version 3.18.1) in R (version 4.0.5) (Yu et al., OMICS, 2012).

For manuscripts utilizing custom algorithms or software that are central to the research but not yet described in published literature, software must be made available to editors and reviewers. We strongly encourage code deposition in a community repository (e.g. GitHub). See the Nature Portfolio guidelines for submitting code & software for further information.

Data

Policy information about availability of data

All manuscripts must include a data availability statement. This statement should provide the following information, where applicable:

- Accession codes, unique identifiers, or web links for publicly available datasets
- A description of any restrictions on data availability
- For clinical datasets or third party data, please ensure that the statement adheres to our policy

Data availability: RNA-seq and Methyl-seq data have been deposited with ArrayExpress database (https://www.ebi.ac.uk/biostudies/arrayexpress) accession numbers E-MTAB-10280 and E-MTAB-10281. The Arabidopsis reference genome (TAIR10 version 47) and gene annotation are publicly available at http://ftp.ensemblgenomes.org/pub/plants/release-47/. Araport11 annotation for transposons and pseudogenes are publicly available at https://datacommons.cyverse.org/browse/iplant/home/araport/public_data/Araport11_Release_201606/annotation. The authors declare that all other data supporting the findings of this study are available in the main text or the supplementary materials.

Field-specific reporting

Please select the one below that is the best fit for your research. If you are not sure, read the appropriate sections before making your selection.

☒ Life sciences ☐ Behavioural & social sciences ☐ Ecological, evolutionary & environmental sciences

For a reference copy of the document with all sections, see nature.com/documents/nr-reporting-summary-flat.pdf

Life sciences study design

All studies must disclose on these points even when the disclosure is negative.

Sample size	There was no specific statistical method used to determine sample size, except for the number of leaves for INTACT experiments and the numbers of nuclei used for RNA-seq, Stem-loop qPCR and EM-seq experiments. These numbers were determined experimentally. Approximately 650 leaves of eINTACT-reporter plants inoculated with Xcc*AvrBs3 or Xcc ΔxopD*AvrBs3 were required for yielding ~250,000 nuclei. The obtained nuclei were divided into ~100,000 nuclei for RNA-seq, ~100,000 nuclei for Stem-loop qPCR and ~50,000 nuclei for EM-seq, to extract enough nuclear RNA or DNA used as starting materials for these experiments.<br>For other experiments, we used sample sizes that are required for yielding sufficient statistical power in previous studies such as in You et al., Nature Communications (2017); You et al., Plant Cell (2019): Guy et al., mBio (2013); Peng et al., PNAS (2019); Manavella et al., Cell (2012).
Data exclusions	No data were excluded from the analyses.
Replication	All experiments contain 2-4 independent biological replicates. The Illumina sequencing and LC-MS experiments were performed once using 3-4 independent biological replicates of each sample with 2-3 technical replicates per sample. All other experiments were repeated at least twice independently to verify the reproducibility of the findings. All the replicates were successful.
Randomization	Plants were always randomly distributed during growth and after treatment. Samples were harvested randomly for data collection.
Blinding	We are blinded to sample group allocation during all manual data collection and analysis, including recording disease indexes in leaves infected by different bacterial strains and phenotypes after different chemical treatments, measurement of water loss rate etc.<br>Blinding was not necessary for the automatic data collection and processing using machines or software, including imaging, quantitative PCR and Illumina sequencing analyses etc.

Reporting for specific materials, systems and methods

We require information from authors about some types of materials, experimental systems and methods used in many studies. Here, indicate whether each material, system or method listed is relevant to your study. If you are not sure if a list item applies to your research, read the appropriate section before selecting a response.

Materials & experimental systems

n/a	Involved in the study	
☐	☒	Antibodies
☒	☐	Eukaryotic cell lines
☒	☐	Palaeontology and archaeology
☐	☒	Animals and other organisms
☒	☐	Human research participants
☒	☐	Clinical data
☒	☐	Dual use research of concern

Methods

n/a	Involved in the study	
☒	☐	ChIP-seq
☒	☐	Flow cytometry
☒	☐	MRI-based neuroimaging

Antibodies

Antibodies used	A rabbit polyclonal anti-H3 antibody (Millipore, Cat.17–10254, Lot. 2051404).<br>An IRDye® 680RD Donkey anti-Rabbit IgG secondary antibody (LI-COR, Cat.925-68073).<br>A mouse monoclonal (Clone no. B-2), HRP-conjugated anti-GFP antibody (Santa Cruz Biotechnology, Cat. sc-9996 HRP)
Validation	The anti-H3 antibody was validated to specifically detect H3 protein in Arabidopsis thaliana plants (You et al., Nat Commun, 2017).<br>The anti-GFP has been used to detect GFP and GFP-fusion proteins in mammalian species in a variety of scientific publications (Santa Cruz Biotechnology, https://datasheets.scbt.com/sc-9996.pdf) and in Arabidopsis thaliana plants (Zhang et al., ScienceAdvances, 2022, DOI: 10.1126/sciadv.abn5488).

Animals and other organisms

Policy information about studies involving animals; ARRIVE guidelines recommended for reporting animal research

Laboratory animals	The study did not involve laboratory animals.
Wild animals	The study did not involve wild animals.
Field-collected samples	The study did not involve samples collected from the field.
Ethics oversight	No ethical approval or guidance was required.

Note that full information on the approval of the study protocol must also be provided in the manuscript.
